# Advances in research on the mechanisms of anti-inflammation of silybin: a review

**DOI:** 10.3389/fimmu.2026.1742733

**Published:** 2026-03-30

**Authors:** Yang Wang, Yongli Yang

**Affiliations:** Department of Gynecology, Affiliated Hospital of Qinghai University, Xining, Qinghai, China

**Keywords:** anti-inflammatory, mechanisms, NF-κB, oxidative stress, silybin

## Abstract

Silybin, as the main active component of milk thistle, has attracted significant attention in recent medical research due to its remarkable anti-inflammatory effect. Inflammatory responses are an important mechanism in the pathogenesis of various clinical diseases, and controlling inflammation is crucial for the prevention and treatment of diseases. Existing studies have shown that silybin can regulate the release of inflammation-related cell factors, inhibit oxidative stress responses, modulate apoptosis, and immune function, thereby exerting multi-target and multi-pathway anti-inflammatory effects. This article systematically reviews the anti-inflammatory mechanisms of silybin in various clinical diseases, including neurological diseases, respiratory diseases, and digestive system diseases, and analyzes the latest progress in its mechanisms of action, aiming to provide a theoretical basis for its clinical applications and promote the development and application of silybin as a novel anti-inflammatory drug.

## 1 Introduction

**Figure 1:**
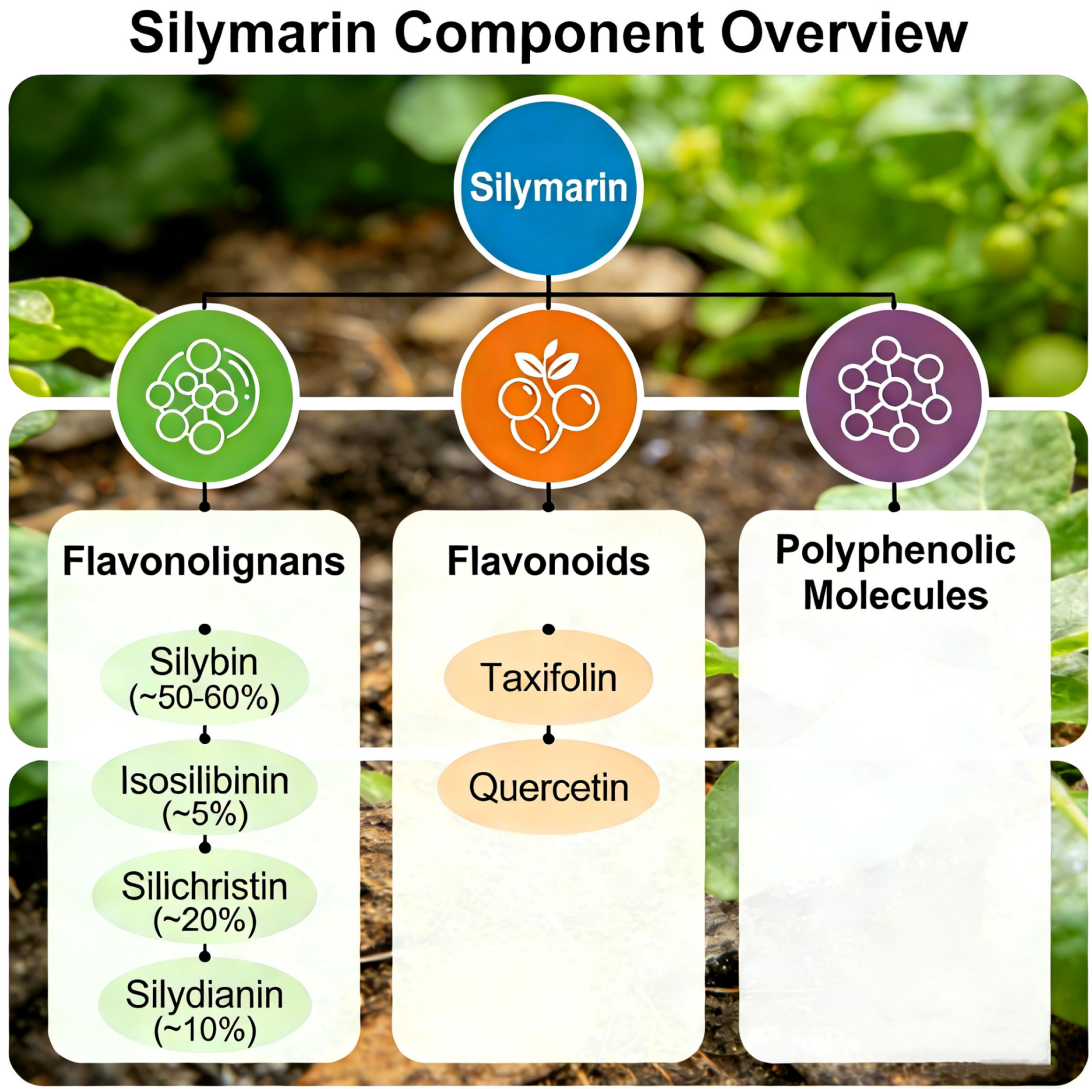
Major components of silymarin. Silymarin, an extract from Silybum marianum, consists mainly of flavonolignans (silybin, ~50–60%; isosilybinin, ~5%; silichristin, ~20%; silydianin, ~10%) and flavonoids (taxifolin, quercetin), which underpin its biological effects.

**Figure 2:**
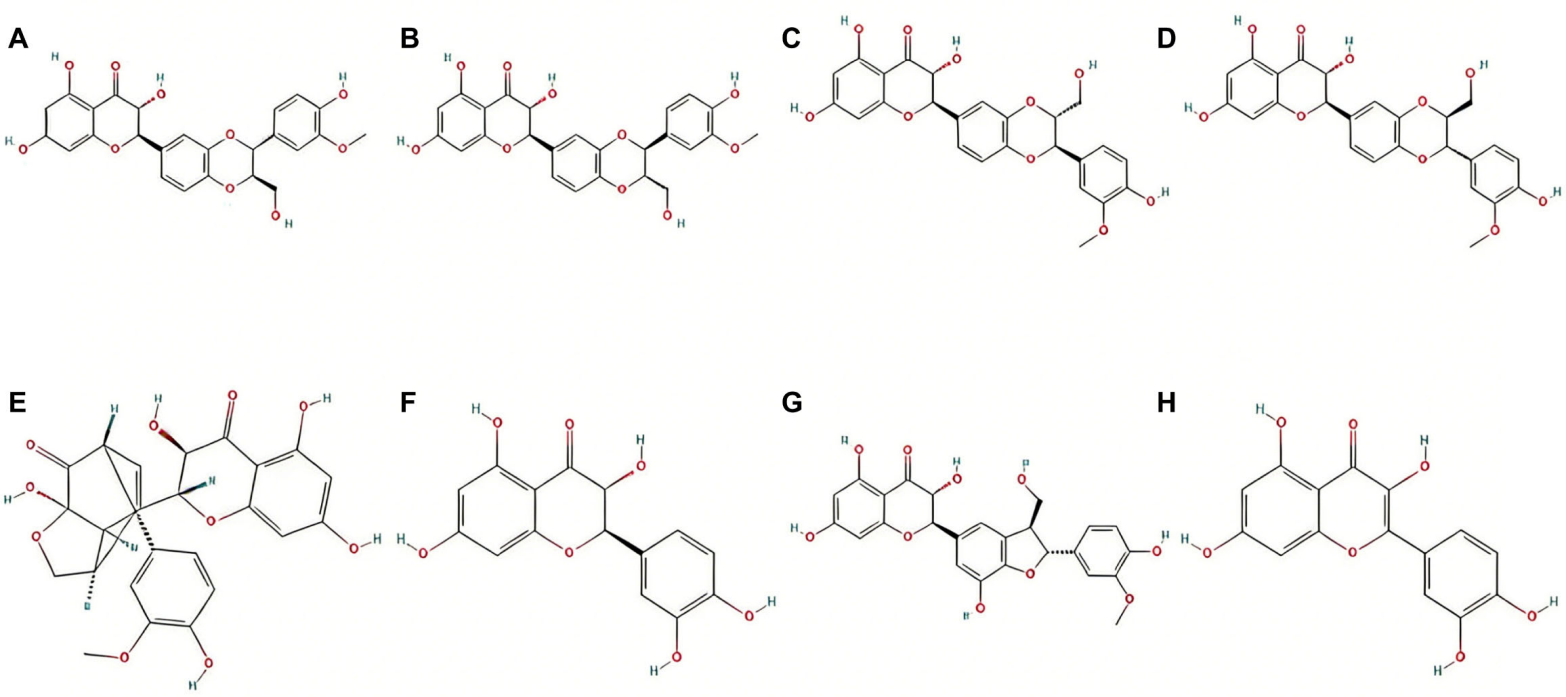
Chemical structures of the main bioactive components in silymarin. **(A)** silybin A, **(B)** silybin B, **(C)** isosilybin A, **(D)** isosilybin B, **(E)** silydianin, **(F)** taxifolin, **(G)** silychristin, and **(H)** quercetin.

Silybin, as the main active component of milk thistle, is a natural flavonoid that has garnered significant attention for its outstanding pharmacological activities. It is primarily found in silymarin, which is a mixture of polyphenolic flavonoids extracted from the seeds and fruits of milk thistle and includes silybin A, silybin B, and isosilybin, among others (**Figure 1**) (1, 2). **Figure 2** shows the chemical structures of the main components in silymarin (3). These components collectively confer upon milk thistle notable antioxidant, anti-inflammatory, antiviral, and anti-tumor properties. Given its anti-inflammatory effects, silybin has become a key research subject for treating various systemic diseases, particularly those of the liver, nervous system, and cardiovascular system (4, 5).

Inflammation is the pathological basis of various diseases, involving complex cellular signaling and immune regulation mechanisms. The inflammatory response is not only the body’s defense reaction to pathogens and injuries, but also, in a chronic inflammatory state, can lead to tissue injury and disease progression (6, 7). Controlling the inflammatory response, especially inhibiting excessive immune activation and cytokine release, is a key aspect of treating multi-system diseases, including autoimmune diseases, metabolic syndrome, and neurodegenerative diseases (8, 9). This context underscores the growing interest in natural multi-target anti-inflammatory compounds. Studies indicate that silybin exerts potent anti-inflammatory efficacy by regulating immune cell functions, inhibiting pro-inflammatory factor release, and modulating key signaling pathways such as NF-κB and mitogen-activated protein kinase (MAPK) (10, 11).

Owing to its multi-level immune regulatory capabilities, silybin has shown clear therapeutic potential in experimental models of various systemic diseases. For example, in animal models of multiple sclerosis, silybin can inhibit the activation of dendritic cells and the differentiation of Th17 cells, significantly reducing inflammation and demyelination in the central nervous system (8). In models of intracerebral hemorrhage, it promotes the polarization of microglia toward the protective M2 phenotype, suppresses pro-inflammatory factor expression, and reduces neuronal apoptosis, collectively exerting neuroprotective effects (11). In liver diseases, silybin not only alleviates oxidative stress damage through its antioxidant effects but also regulates immune cell infiltration and inflammatory signaling pathways, slowing the progression of liver fibrosis and hepatocellular carcinoma (12, 13). Furthermore, silybin has been shown to regulate NAD+ metabolism and SIRT2 expression while inhibiting the NF-κB pathway, mechanisms that help restore hepatic metabolic enzyme function and mitigate inflammation in nonalcoholic fatty liver disease (14). In the realm of cardiovascular and metabolic diseases, silybin exerts beneficial effects on atherosclerosis, hypertension, and diabetic complications by improving vascular endothelial inflammation, regulating lipid metabolism, and ameliorating insulin resistance (15–17). Notably, silybin exhibits dual anti-proliferative and immunomodulatory effects in oncology. It can inhibit tumor cell proliferation, induce apoptosis, and modulate the immune response within the tumor microenvironment, thereby achieving a synergistic anti-inflammatory and anti-tumor effect (18, 19).

In summary, silybin, as a natural flavonoid with multi-target anti-inflammatory activity, shows broad application prospects in treating inflammation-related multi-system diseases. It alleviates pathological conditions and promotes tissue repair by regulating immune and inflammatory responses through multiple levels and pathways. Looking forward, silybin is expected to become an important candidate drug for anti-inflammatory therapy, particularly when combined with novel drug delivery systems and precise molecular mechanism studies. This article will systematically review the anti-inflammatory mechanisms of silybin in various systemic diseases, integrating the latest fundamental and clinical research to explore its clinical translation potential and future research directions.

## 2 The chemical structure and biological activity of silybin

### 2.1 Chemical structure

Silybin is a flavonoid lignan extracted from milk thistle, with a complex chemical structure that can be subdivided into three main regions (13). The core skeleton of silybin consists of a dihydroflavonol moiety (ring A), a phenylpropanoid moiety (ring B), and a benzodioxole connecting bridge linking the two (20). The molecule contains multiple phenolic hydroxyl groups, which are key sites for its biological activity. Research shows that the hydroxyl groups at positions 5 and 7 of ring A, as well as the hydroxyl groups at positions 3’ and 4’ of ring B, together form a phenolic hydroxyl network, which is crucial for its anti-oxidation and interaction with targets (21). The stereochemistry of the connecting bridge, especially the configuration at C-2 and C-3, as well as the structure of the benzodioxole itself, imparts rigidity to the molecule and profoundly affects its biological activity, making this region a site that requires careful handling in structural modifications. For example, the derivatives obtained by methylation of the hydroxyl groups at positions 5, 7, and 20 showed significant changes in anti-proliferative activity compared to the parent compound, which directly demonstrates the importance of these functional groups. In addition, silybin exists in two non-enantiomeric isomers, silybin A and silybin B, coexisting in a molar ratio close to 1:1, with different stereoconfigurations at C-10 and C-11, and this stereochemical difference also leads to differences in biological activity (22–24).

### 2.2 The pharmacophore basis of anti-inflammatory activity

The anti-inflammatory activity of silybin is closely related to its specific pharmacophore characteristics. Among them, the phenolic hydroxyl network is the core pharmacophore that exerts its anti-oxidation, scavenges free radicals, and directly regulates kinase activity (21). In particular, the ortho-dihydroxy group (catechol structure) at the 3’, 4’-positions on ring B is crucial for inhibiting the activation of NF-κB, as this structure can form hydrogen bonds or covalent bonds with key proteins in the signaling pathways, thereby blocking the transmission of inflammatory signals. Research shows that silybin combined with thymol can synergistically inhibit the activation of NF-κB and MAPK signaling pathways induced by lipopolysaccharides in RAW264.7 macrophages, and this synergistic effect is partly attributed to the interactions of these key phenolic hydroxyl groups (25). In addition to the hydrophilic regions, the hydrophobic parts of the molecule, such as the methoxy and phenyl rings on the phenylpropanoid unit, affect its interaction with cell membranes and protein targets (20). The overall planarity and hydrophobicity of the molecules determine their membrane translocation ability and distribution within the cells. For example, by linking silybin with L-carnosine through a phosphodiester bond to form a novel bioconjugate, not only is the solubility of silybin improved, but also its key pharmacophore is retained, thereby exhibiting superior antioxidant and cytoprotective effects compared to the parent compound *in vitro* experiments (21). These structural features together constitute the basis for the interaction of silybin with inflammation-related targets, such as cyclooxygenase-2 (COX-2), phosphoinositide 3-kinase gamma (PI3Kγ), etc., which is the molecular basis for its multi-pathway anti-inflammatory effects (25, 26).

### 2.3 Biological activity

Silybin is extracted from the fruits and seeds of milk thistle. Its chemical structure is a hybrid of flavonoid and lignan motifs, containing multiple active sites amenable to chemical modification. Notably, it exists as distinct stereochemical isomers. These characteristics endow silybin with great potential in treating various diseases through anti-inflammatory, antioxidant, and anticancer mechanisms. A deep understanding of its chemical structural characteristics, in comparison with other flavonoids, can help guide the design and optimization of its derivatives, thereby enhancing its clinical application value.

The anti-inflammatory effect of silybin primarily manifests in the inhibition of excessive activation of immune cells and the release of inflammatory factors. For example, in a murine model of multiple sclerosis (experimental autoimmune encephalomyelitis, EAE), silybin significantly alleviated disease severity and improved neuroinflammation and demyelination in the central nervous system by inhibiting dendritic cell activation and Th17 cell differentiation (8), indicating its potential therapeutic value for autoimmune diseases. Mechanistically, its anti-inflammatory activity involves the regulation of multiple signaling pathways: Silybin can inhibit pathways such as PI3K/AKT/NF-κB, thereby regulating the expression of inflammatory mediators like nitric oxide (NO), TNF-α, and IL-6 (27–29). Recent studies further confirm that silybin can exert protective effects by reducing the inflammatory response and cell death through the inhibition of Pannexin1 channel activity (30). In various *in vivo* and *in vitro* models, silybin has shown protective effects against inflammation-related diseases, including respiratory and neurodegenerative diseases (31, 32).

Beyond its anti-inflammatory role, the antioxidant properties of silybin are also crucial to its biological functions. It can effectively reduce the generation of reactive oxygen species (ROS), alleviate oxidative stress, enhance the activity of intracellular antioxidant enzymes, and inhibit oxidative damage. For example, in an *in vitro* 3T3-L1 adipocyte model, silybin can significantly reduce TNF-α and improve glucose uptake, and this effect is unrelated to GLUT 4 protein expression (27). Research has also found that when encapsulated in polycaprolactone electrospun fiber membranes, silybin effectively protects cells against oxidative stress, demonstrating good antioxidant potential and biocompatibility (33).

Regarding anti-tumor effects, the mechanisms of action of silybin and its derivatives are diverse, including inhibiting cancer cell proliferation, inducing apoptosis, inhibiting tumor-related signaling pathways, and regulating immune responses. For example, silybin’s dual inhibitory effect on BRAF and SMO receptors in melanoma cells provides new targets for anti-tumor therapy (34). Additionally, the silybin derivative compound 15k exhibits significantly stronger anti-proliferative and anti-tumor activities against various cancer cells, such as ovarian cancer and breast cancer, compared to the parent compound silybin. By targeting and binding to α-tubulin, inducing sub-G1 phase cell cycle arrest and apoptosis, it emerges as a promising novel anti-tumor candidate molecule (35).

## 3 The application of silybin in neurological diseases

### 3.1 The impact on Alzheimer’s disease

Research has found that silybin exerts notable anti-inflammatory and neuroprotective effects in Alzheimer’s disease, primarily by inhibiting the excessive activation of microglia induced by amyloid-β (Aβ). At the molecular level, silybin binds with submillimolar affinity to Aβ oligomers, using its A ring as the primary binding site. This interaction stabilizes the oligomer conformation and shields the toxic surfaces of the N-terminal and central hydrophobic core of Aβ monomers. Consequently, silybin dose-dependently inhibits the aggregation of Aβ_40_/Aβ_42_ and reduces the formation of toxic aggregates. Furthermore, its phenolic hydroxyl network enables the scavenging of reactive oxygen species, thereby alleviating the vicious cycle of “oxidative stress-neuroinflammation” and protecting neurons from the toxicity of Aβ oligomers. Notably, the silybin B isomer exhibits greater activity than silybin A, and structural modification with fucose can enhance water solubility and serum stability, which prolongs the duration of its anti-inflammatory effects *in vivo*. Latest animal model studies further confirm the potential of silybin in slowing Alzheimer’s disease progression, revealing that it can not only delay Aβ deposition but also ameliorate cognitive impairment (36).

### 3.2 The impact on Parkinson’s disease

In the pathogenesis of Parkinson’s disease, neuroinflammation and oxidative stress are key factors leading to the loss of dopaminergic neurons in the substantia nigra. Studies demonstrate that silybin can effectively mitigate neuroinflammation by reducing the expression of pro-inflammatory cytokines such as IL-1β, TNF-α, and IFN-β, and by suppressing the activation of the STING-IRF3-IFN-β pathway (37). Furthermore, silybin protects mitochondrial function through enhancing the antioxidant defense system, reducing lipid peroxidation, and promoting neuronal autophagy to clear damaged mitochondria, thereby safeguarding dopaminergic neurons (38, 39). Research also indicates that silybin, which is present in a higher proportion within silymarin B, exhibits a stronger inhibitory effect on α-synuclein amyloid fibril formation. This reduction in toxic protein aggregates may indirectly alleviate neuroinflammatory activation (40). In addition, molecular docking and dynamics simulation studies have identified silybin as a potential effective inhibitor of the P2X7 receptor, suggesting a further mechanism whereby silybin may exert anti-inflammatory and neuroprotective effects by blocking inflammation-mediated cell death pathways (41).

### 3.3 Effect on subarachnoid hemorrhage

Research has found that silybin can improve neuronal injury and short-term neurological function in mice with subarachnoid hemorrhage, reducing inflammatory damage and neuronal apoptosis. Its protective mechanism involves inhibiting the STING signaling pathway, thereby promoting the polarization of microglial cells towards the M2 anti-inflammatory phenotype, reducing the expression of pro-inflammatory factors (such as TNF-α, IL-1β, NLRP3), and increasing the expression of anti-inflammatory factors (such as CD206, Arg1, IL-10), thereby alleviating neuroinflammation and neuronal apoptosis after subarachnoid hemorrhage. Subsequently, it was found that silybin reduced the mRNA and protein levels of the stimulator of interferon genes (STING) in microglia (11).

Silybin has good anti-inflammatory and neuroprotective effects in nervous system diseases such as Alzheimer’s Disease, Parkinson’s disease, and subarachnoid hemorrhage. Its mechanisms mainly focus on inhibiting neuroinflammation, reducing oxidative stress, and protecting neurons (**Table 1**).

**Table 1:** The role of silybin in neurological disorders.

Diseases	Key associated effect	Regulated factors	Reference
Alzheimer’s disease	↓ Excessive activation of microglia induced by Aβ; binds to Aβ oligomers, stabilizes their conformation, and shields the toxic surfaces; ↓ aggregation of Aβ_40_/Aβ_42_;↓ ROS, alleviating the vicious cycle of “oxidative stress–neuroinflammation”; silybin B shows higher activity, fucose modification can improve solubility and stability; delays Aβ deposition and ameliorates cognitive impairment	↓ Aβ, ↓ Aβ_40_/Aβ_42_,↓ ROS, silybin A/silybin B	(36)
Parkinson’s disease	↓ Pro-inflammatory cytokines, ↓ STING-IRF3-IFN-β signaling pathway, alleviate neuroinflammation	↓ IL-1β, ↓ TNF-α, ↓ IFN-β, ↓ STING-IRF3-IFN-β signaling pathway	(37)
	↑ The antioxidant defense system protects mitochondrial function, ↓ lipid peroxidation; ↑ neuronal autophagy, clear damaged mitochondria		(38, 39)
	↓ Amyloid fibril formation of α-synuclein, ↓ a toxic protein aggregation		(40)
	Act as a potential inhibitor of the P2X7 receptor and block inflammation-mediated cell death		(41)
Subarachnoid hemorrhage	↑ Alleviate neuronal injury, improve neurological function, ↓ inflammation and apoptosis; ↓ STING signaling pathway, ↑ polarization of microglia towards the M2 phenotype; ↓ pro-inflammatory cytokines, ↑ anti-inflammatory cytokines; ↓ mRNA and protein levels of STING in microglia	↓ STING signaling pathway;↓ TNF-α, ↓ IL-1β, ↓ NLRP3; ↑ CD206, ↑ Arg1, ↑ IL-10	(11)

“↑” represents silybin’s promoting effect. “↓” represents silybin’s inhibiting effect.

## 4 Application of silybin in respiratory system diseases

### 4.1 The impact on tuberculosis

The characteristics of tuberculosis infection include granulomatous lung injury and a systemic inflammatory response (42). This infection induces the release of various inflammatory mediators, such as tumor necrosis factor-α (TNF-α), interleukin-1β (IL-1β), and IL-6, which participate not only in immune defense but also in the regulation of inflammatory responses (43). In response to Mycobacterium tuberculosis infection, the host immune response plays a critical role. Multiple immune cells—including macrophages, dendritic cells, neutrophils, mast cells, and monocytes—can recognize danger signals and activate the NLRP3 inflammasome, thereby initiating inflammatory responses to eliminate pathogens and promote infection resolution (42). In a study using a mouse model of experimental pulmonary tuberculosis, silybin was found to promote the expression of Th1-type cytokines such as IFN-γ, IL-12, and TNF-α, thereby enhancing the host protective immune response against Mycobacterium tuberculosis (44). The anti-inflammatory mechanism of silybin is multifaceted. In a lipopolysaccharide-induced acute lung injury model in mice, its protective effect does not depend on altering the protein expression levels of NLRP3, ASC (apoptosis-associated speck-like protein containing a CARD domain), or caspase-1. Instead, it is achieved through synergistic inhibition of the functional activity of both the NLRP3 inflammasome and the NF-κB signaling pathway (42). These findings suggest that silybin may exert anti-inflammatory and protective effects by fine-tuning the function of key inflammatory signaling hubs.

### 4.2 Effects on other lung diseases

Many respiratory system diseases share a common pathological basis closely linked to chronic inflammatory responses. The persistence of inflammation leads to airway structural remodeling, dysfunction, and tissue fibrosis, severely affecting patients’ quality of life and survival (45, 46). Silybin shows potential for alleviating various pulmonary inflammatory diseases, with important mechanisms involving the regulation of key inflammatory signaling pathways. For example, research indicates that it can effectively inhibit the activation of the NF-κB signaling pathway and reduce the phosphorylation levels of key proteins in the MAPK signaling pathway.

(47). In the pneumonia model induced by silica dioxide nanoparticles (SiONPs), a key anti-inflammatory mechanism of silybin involves inhibition of the TXNIP/MAPK/AP-1 signaling pathway. Both *in vitro* and *in vivo* experiments demonstrate that silybin can effectively downregulate the expression of thioredoxin-interacting protein (TXNIP) induced by nanoparticle stimulation. This inhibition suppresses the phosphorylation and activation of the downstream MAPK signaling pathway, ultimately reducing the activity of the transcription factor AP-1. This blockade of the signaling cascade results in a significant decrease in the mRNA expression and protein levels of key pro-inflammatory cytokines (such as TNF-α, IL-6, and IL-1β), thereby alleviating inflammatory cell infiltration and tissue damage (48).In the context of pulmonary viral infections such as SARS-CoV-2, silybin demonstrates a multi-target mode of action. On one hand, it can potentially inhibit viral entry and replication by stably binding to the viral spike protein RBD and the main protease Mpro, exerting a direct antiviral effect. On the other hand, at the host level, it significantly inhibits the TNF-α-triggered “cytokine storm” and downregulates the gene expression of key pro-inflammatory mediators (e.g., IL-6, MCP-1). Simultaneously, it reduces factors closely associated with endothelial dysfunction, pathological coagulation (PAI-1), and excessive vasoconstriction (ET-1). Through these integrated actions, silybin alleviates the excessive inflammation, endothelial injury, and thrombotic risk induced by viral infection, highlighting its multifaceted therapeutic potential (49).

Silybin can exert anti-inflammatory and protective effects in respiratory diseases such as pulmonary tuberculosis and pneumonia by promoting the expression of Th1-type cytokines, inhibiting the NF-κB/MAPK pathway, and suppressing the activity of the NLRP3 inflammasome (**Table 2**).

**Table 2:** The role of silybin in respiratory system diseases.

Diseases	Key associated effect	Regulated factors	Reference
Tuberculosis	↑ Expression of Th1-type cytokines, ↑ host protective immune response against Mycobacterium tuberculosis	↑ IFN-γ, ↑ IL-12, ↑ TNF-α	(44)
	↓ Functional activity of the NLRP3 inflammasome and the NF-κB signaling pathway	↓ NLRP3 inflammasome, ↓ NF-κB signaling pathway	(42)
SiONPs-induced pneumonia	↓ TXNIP/MAPK/AP-1 signaling pathway, ↓ mRNA and protein levels of pro-inflammatory cytokines	↓ TXNIP/MAPK/AP-1 signaling pathway, ↓ TXNIP, ↓ MAPK, ↓ AP-1, ↓ TNF-α, ↓ IL-6, ↓ IL-1β	(48)
Viral pneumonia	Bind to the viral spike protein RBD and the main protease Mpro, ↓ viral entry and replication;↓ TNF-α-mediated cytokine storm,↓ gene expression of pro-inflammatory mediators;↓ the levels of factors associated with endothelial dysfunction, pathological coagulation, and excessive vasoconstriction; ↓ virus-induced excessive	↓ IL-6, ↓ MCP-1; ↓ PAI-1, ↓ ET-1	(49)
	inflammation, endothelial injury, and thrombotic risk		

“↑” represents silybin’s promoting effect. “↓” represents silybin’s inhibiting effect.

## 5 The application of silybin in cardiovascular diseases

The inflammatory mechanisms of cardiovascular diseases not only participate in the formation of atherosclerosis but also affect various pathological processes such as myocardial ischemia and heart failure. For example, the activation of inflammasomes, especially the NLRP3 inflammasome, leads to endothelial dysfunction, plaque formation, and myocardial injury by inducing the release of pro-inflammatory cytokines such as IL-1β, IL-18, and IL-6 (50, 51). In addition, immune cells (such as macrophages and lymphocytes) play an important role in the inflammation response, which can regulate the inflammatory status of the cardiovascular microenvironment, thereby affecting the disease progression (52, 53). Clinical trials have also confirmed that anti-inflammatory therapy targeting these inflammatory pathways can effectively reduce the risk of cardiovascular events (51, 54).

### 5.1 The anti-inflammatory effect in atherosclerosis

Silybin has been shown to reduce the inflammatory response in atherosclerotic lesions, with its core mechanism involving the direct inhibition of key pro-inflammatory signaling pathways. Specifically, by suppressing the activation of nuclear factor κB (NF-κB), it can effectively downregulate the expression of a series of downstream pro-inflammatory cytokines (such as IL and TNF-α). Simultaneously, silybin improves endothelial dysfunction—a critical link in the onset of inflammation—by lowering asymmetric dimethylarginine (ADMA) levels, thereby reversing nitric oxide synthase inhibition and increasing the bioavailability of NO, which possesses anti-inflammatory and vasodilatory functions. Furthermore, the anti-inflammatory effects of silybin may synergize with the activation of antioxidant pathways such as nuclear factor erythroid 2-related factor 2 (Nrf2) (55). In addition to the aforementioned direct mechanisms acting on blood vessels, studies have also found that silybin can exert anti-inflammatory effects by regulating the “gut microbiota-metabolite-host” axis. The core mechanism is to enhance the levels of butyryl-CoA transferase (BUT) in the gut to promote the production of butyrate, and synergistically induce the expression of butyrate transport proteins (MCT1/MCT4), thereby increasing the entry of butyrate into the bloodstream. Butyrate entering the circulation can directly improve endothelial function and inhibit inflammation; at the same time, the elevated levels of butyrate mediated by silybin can enhance intestinal tight junctions and repair barrier integrity, thereby inhibiting systemic inflammatory responses caused by increased intestinal permeability, and alleviating atherosclerosis through multiple pathways (56). In terms of therapeutic applications, in a diabetic atherosclerosis model, the combination of silybin and clopidogrel can significantly reduce lesions, inflammation, and endothelial dysfunction at the aortic root. Experimental data indicate that the combination therapy can more effectively inhibit vascular wall inflammatory responses, which may be achieved through a comprehensive approach that improves diabetic conditions, alleviates endothelial damage, and synergistically regulates platelet activity, thereby playing a key anti-inflammatory role in inhibiting the progression of atherosclerosis (57).

### 5.2 The protective mechanisms in hypertension, myocardial infarction, and ischemia-reperfusion injury

The occurrence and development of hypertension are closely related to vascular inflammation and vascular remodeling. The endothelial cells, smooth muscle cells, and immune cells of the vessel wall can form a complex inflammatory network by secreting inflammatory mediators, which jointly promote the sustained increase of blood pressure (58, 59). Molecular docking predictions and functional experiments confirm that silybin can bind to and activate the TRPV4 channel, inducing calcium ion influx in endothelial cells, leading to endothelium-dependent vasodilation. In a high-salt-induced hypertensive mouse model, silybin lowers blood pressure and improves vascular relaxation response to acetylcholine through this pathway (15).

Beyond its impact on hypertension, silybin also exhibits a protective effect on heart failure after myocardial infarction. Its core anti-inflammatory mechanism is closely related to the targeted inhibition of the Hypoxia-Inducible Factor 1-alpha (HIF-1α) signaling pathway, thereby correcting pathological glycolytic metabolism. Specifically, in both *in vivo* and *in vitro* models, silybin can effectively inhibit the expression and nuclear translocation of HIF-1α, subsequently downregulating the expression of key glycolytic regulatory proteins (such as PFKFB3, GLUT1, LDHA) downstream. This action directly reduces excessive glycolysis, lactate production, and reactive oxygen species accumulation in cardiomyocytes. Consequently, it alleviates myocardial inflammation, fibrosis, and dysfunction driven by energy disruption and oxidative stress from the metabolic source, ultimately improving cardiac remodeling and function. Therefore, silybin exerts a crucial cardiovascular anti-inflammatory and protective effect through HIF-1α-mediated metabolic reprogramming (60).

Silybin can exert anti-inflammatory, anti-fibrotic, and cardioprotective effects in cardiovascular diseases such as atherosclerosis, hypertension, and myocardial infarction through mechanisms such as regulating lipid metabolism, repairing the gut barrier, improving vascular dilation function, correcting myocardial energy metabolism disorders, and inhibiting inflammatory pathways (**Table 3**).

**Table 3:** The role of silybin in cardiovascular diseases.

Diseases	Key associated effect	Regulated factors	Reference
Atherosclerosis	↓ Activation of the NF-κB signaling pathway; ↓ expression of downstream pro-inflammatory cytokines; ↓ ADMA levels, endothelial function, ↑ bioavailability of NO; ↑ synergistic anti-inflammatory effect exerted by the Nrf2 antioxidant pathway	↓ NF-κB signaling pathway; ↓ ADMA; ↑ Nrf2 signaling pathway	(55)
	Regulate the gut microbiota-metabolite-host axis;↑ butyrate production and transport, and repair the intestinal barrier	↑ BUT, ↑ MCT1/MCT4	(56)
	When combined with clopidogrel, it alleviates aortic root lesions, inflammation, and endothelial injury in a diabetic atherosclerosis model.		(57)
Hypertension	Bind to and activate the TRPV4 channel, induce calcium influx in endothelial cells, and mediate vasodilation; reduce blood pressure and improve vasodilatory response.	↑ TRPV4 channel, ↑ Ca²^+^ influx	(15)
Myocardial infarction/ischemia-reperfusion injury/heart failure	↓ HIF-1α expression and nuclear translocation; ↓ key glycolytic regulatory proteins, alleviate myocardial inflammation, fibrosis, and cardiac dysfunction	↓ HIF-1α signaling pathway, ↓ PFKFB3, ↓ GLUT1, ↓ LDHA	(60)

“↑” represents silybin’s promoting effect. “↓” represents silybin’s inhibiting effect.

## 6 The application of silybin in digestive system diseases

### 6.1 The impact on liver fibrosis

Silybin has received significant attention in recent years for its remarkable anti-oxidation, anti-inflammatory, and anti-fibrosis effects in the treatment of liver diseases. Hepatic fibrosis is a common pathologic feature of various liver diseases, and its occurrence and development are closely related to hepatocyte injury and inflammatory responses.

Firstly, focusing on antioxidant and metabolic regulatory mechanisms, silybin can activate the Nrf2 signaling pathway, enhancing the expression of intracellular antioxidant enzymes (such as superoxide dismutase and glutathione), effectively clearing reactive oxygen species (ROS) and alleviating oxidative stress damage (61). For example, silybin derivative A2 has shown excellent antioxidant activity in a model of metabolic-associated fatty liver disease, inhibiting lipid accumulation and liver inflammation, significantly improving the pathological features of hepatic fibrosis (62). Beyond direct antioxidant effects, silybin can also exert anti-hepatic fibrosis effects by targeting and regulating mitochondrial metabolism and succinate homeostasis. The core mechanism involves repairing mitochondrial membrane phospholipids and upregulating CRLS1 to promote the assembly and activity of the succinate dehydrogenase complex (SDHA/SDHB), thereby reducing succinate accumulation within mitochondria. Simultaneously, it downregulates the transporter protein MCT1 to inhibit the release of succinate into the extracellular space. The reduced extracellular succinate levels further inhibit the activation of hepatic stellate cells (LX-2), thus blocking the inflammatory and fibrotic processes from the metabolic source (63).

In terms of modulating inflammation signaling within the liver, conditioned medium from Kupffer cells treated with a high-fat diet or palmitic acid promotes liver inflammation. Silybin can counteract this by activating the deacetylase SIRT2 (likely through increasing intracellular NAD+ levels via downregulating PARP-1 expression). The activated SIRT2 then deacetylates the key subunit p65 of NF-κB, inhibiting its nuclear translocation and thereby effectively blocking NF-κB-mediated pro-inflammatory signaling. Functionally, this anti-inflammatory effect is associated with reversing the inflammation-induced inhibition of the drug-metabolizing enzyme CYP3A, restoring its expression and activity (14). A central cellular target in hepatic fibrosis is the hepatic stellate cell (HSC). Silybin and its formulations (such as Silybin methylamine, SLB-M) primarily inhibit the activation and proliferation of HSCs directly, thereby blocking key links in inflammation and fibrosis. Concurrently, they improve the hepatic inflammatory environment by regulating bile acid (BA) metabolic homeostasis. At the molecular level, this action involves the activation of the Farnesoid X receptor (Fxr), which has direct anti-fibrotic properties and can regulate the expression of genes related to BA synthesis and transport (such as upregulating Cyp7a1, Sult2a8, and downregulating Slc51b/OSTβ). Network pharmacological analysis further indicates that its targets are significantly enriched in inflammation-related signaling pathways such as IL-6. In addition, its role in alleviating liver oxidative stress also has an indirect effect on the inflammatory state (64). Supporting its translational relevance, compound preparations containing silybin have shown reductions in inflammatory markers (such as high-sensitivity C-reactive protein) in clinical settings, suggesting its potential anti-inflammatory effect in non-alcoholic fatty liver disease (NAFLD)-related hepatic fibrosis (65).

In the context of metabolic disease-related liver injury, the anti-inflammatory mechanism of silybin-loaded liposomes (SLB) in a rat model of type 2 diabetes combined with NAFLD primarily centers on the activation of the AMPK pathway, which in turn dually inhibits metabolic disorders and pro-fibrotic signals. SLB enhances the phosphorylation level of AMPK in the liver, which improves overall glucose and lipid metabolic disorders, thereby reducing inflammation triggered by lipid accumulation at the source. On the other hand, it significantly downregulates the expression of the pro-fibrotic key factor TGF-β1 and the phosphorylation of its downstream Smad2/3, thereby directly inhibiting the processes of liver inflammation and fibrosis (manifested as a reduction in collagen I/III and α-SMA). This AMPK-mediated inhibition of the TGF-β1/Smad signaling pathway is reversed by the AMPK inhibitor Compound C in hepatocyte models, confirming the central role of this pathway (66).

**Figure 3:**
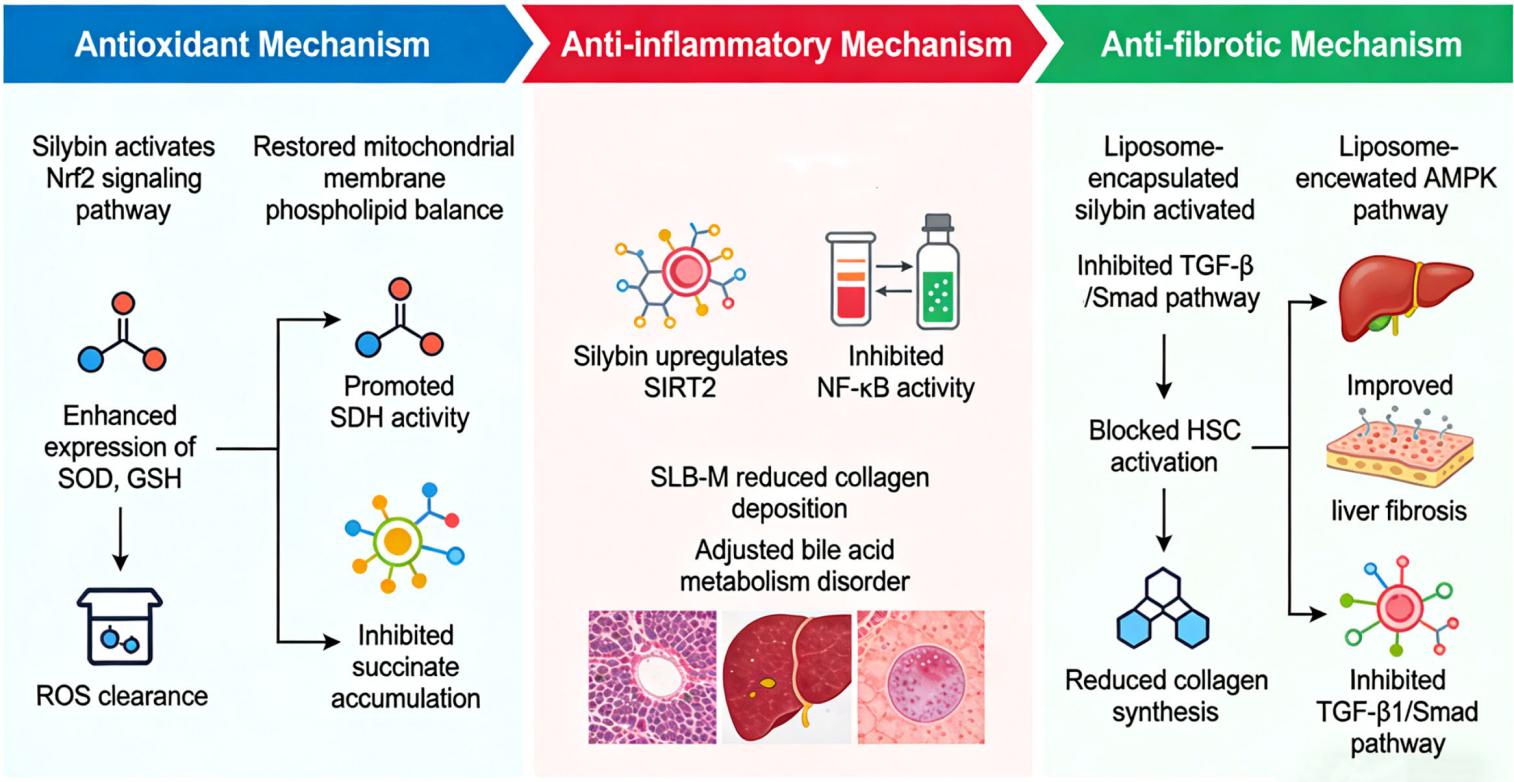
Schematic representation of the multifaceted anti-fibrosis mechanisms of silybin. The illustration delineates the antioxidant, anti-inflammatory, and anti-fibrotic pathways modulated by silybin. The antioxidant mechanism is mediated through the activation of the Nrf2 signaling pathway, leading to the clearance of reactive oxygen species (ROS), enhanced expression of antioxidant enzymes (SOD, GSH), and promotion of SDH activity. This cascade contributes to restoring mitochondrial membrane phospholipid balance and inhibiting succinate accumulation. The anti-inflammatory mechanism involves the upregulation of SIRT2, which in turn inhibits NF-κB activity, reduces collagen deposition, and ameliorates bile acid metabolism disorders. The anti-fibrotic mechanism, particularly enhanced by liposome-encapsulated silybin, operates via the inhibition of the TGF-β/Smad pathway and blockade of hepatic stellate cell (HSC) activation, resulting in reduced collagen synthesis. Additionally, activation of the AMPK pathway by liposomal silybin contributes to the overall improvement of liver fibrosis, further suppressing the TGF-β1/Smad pathway.

Furthermore, silybin can disrupt the vicious cycle of liver fibrosis by regulating intercellular interactions. For instance, research has loaded silybin into glycyrrhetinic acid (GA) modified nanoparticles (GA-NPs/SIB), utilizing GA’s high affinity for hepatocytes to achieve targeted delivery. Its core anti-inflammatory mechanism lies in effectively alleviating oxidative stress within hepatocytes, thereby repairing hepatocyte dysfunction caused by the fibrotic process (67). In the inflammatory microenvironment, activated liver cells release reactive oxygen species (ROS) extracellularly, which can diffuse into hepatic stellate cells (HSCs), promoting autophagy and thereby activating HSCs, which in turn drives fibrosis and hepatic carcinoma progression. The antioxidant properties of silybin can systematically eliminate ROS in the liver microenvironment, thereby cutting off this key pathway driving HSC activation. Notably, in a therapeutic combination approach, in a carbon tetrachloride-induced mouse model, combining silybin with GS-9973 (a drug that specifically inhibits SYK kinase in HSCs) produces a synergistic effect, more effectively inhibiting HSC activity, alleviating hepatic fibrosis, and preventing hepatic carcinoma. This provides a key mechanistic basis for its use as a combination therapy strategy (**Figure 3**) (68).

### 6.2 The impact on intestinal diseases

#### 6.2.1 The impact on gastritis

Experiments have found that silybin can reduce gastric mucosal inflammation and improve pathological morphology (such as dysplasia and hyperplasia) in a Helicobacter pylori (H. pylori)-induced gastritis model. Silybin can inhibit the nuclear translocation of the NF-κB p65 subunit and its DNA-binding activity induced by Helicobacter pylori infection. It also inhibits the phosphorylation and nuclear translocation of STAT3. Subsequently, it was discovered that silybin may disrupt the interaction between NF-κB p65 and phosphorylated STAT3 (P-STAT3), thereby blocking their synergistic effect in promoting inflammation. By inhibiting the upstream NF-κB and STAT3 signaling pathways, silybin ultimately effectively downregulates the expression of key downstream inflammatory mediators, COX-2, and inducible nitric oxide synthase (iNOS) (69).

#### 6.2.2 The impact on ulcerative colitis

In a dextran sulfate sodium-induced ulcerative colitis mouse model, silybin alleviates colitis by remodeling the gut microbiome and promoting the production of the key metabolite (R)-2,3-dihydroxyisovaleric acid by beneficial bacteria. This metabolite targets the colonic γ-aminobutyric acid transporter 3 (GAT-3), initiating the GAT-3/RARβ/RORγt signaling axis, ultimately inhibiting the differentiation of pro-inflammatory Th17 cells (70).

#### 6.2.3 The impact on intestinal tumors

Recent studies have found that silybin has different regulatory effects on normal intestinal epithelial cells and intestinal tumor cells, showing protective effects on healthy cells and inhibitory effects on tumor cells (18).

In normal intestinal epithelial cells (such as the IPEC-1 cell line), silybin can promote cell metabolic activity and proliferation, protect the integrity of the mitochondrial membrane, thereby exerting a cytoprotective effect. This protective effect is also reflected in the increased gene expression of the anti-inflammatory effector TGF-β and the decreased expression of the pro-inflammatory factor TNF-α, demonstrating immunomodulatory and protective effects. By contrast, in human colorectal adenocarcinoma CaCo-2 cells, silybin exhibits a significant inhibitory effect on metabolic activity and cell proliferation, while inducing apoptosis and significantly downregulating the expression of various pro-inflammatory factors, including IL-1, IL-6, and TGF-β. This indicates that silybin not only has anti-inflammatory effects but also promotes tumor cell apoptosis and inhibits growth by suppressing the inflammatory microenvironment. This selective mechanism of action provides a theoretical basis for silybin as an adjunctive therapy for colon cancer, suggesting its potential application value in the prevention and treatment of intestinal tumors (18).

Beyond its direct cellular effects, research on drug delivery systems has highlighted another strategy to enhance its efficacy. It has been found that the complex of silybin and 2-hydroxypropyl-β-cyclodextrin (HP-β-CD) (SHβCD) not only enhances the solubility and intestinal permeability of silybin but also restores the intestinal flora imbalance induced by a high-fat diet and improves intestinal barrier integrity, thereby reducing the transmission of intestinal inflammatory signals to the liver at their source. At the hepatic level, SHβCD exhibits stronger anti-lipid accumulation, antioxidant, and anti-inflammatory effects than silybin alone; transcriptomic analysis further suggests that its improvement of liver inflammation and energy homeostasis is a potential mechanism of action. Therefore, this complex, through the dual regulation of drug absorption and intestinal microecology by cyclodextrin, synergistically amplifies the inherent liver-protective and anti-inflammatory effects of silybin (71).

Silybin exerts anti-inflammatory and anti-fibrosis effects by activating Nrf2, inhibiting NF-κB, and suppressing TGF-β/Smad, and shows protective effects on normal cells and selective inhibition of tumor cells in intestinal experiments (**Table 4**).

**Table 4:** The role of silybin in digestive system diseases.

Diseases	Key associated effect	Regulated factors	Reference
Liver fibrosis	↑Nrf2 signaling pathway; ↑ expression of antioxidant enzymes; ↓ and oxidative stress	↑ Nrf2 signaling pathway, ↑SOD, ↑GSH,↓ ROS	(61)
	Repair mitochondrial membrane phospholipids; ↓ succinate accumulation; ↓ and hepatic stellate cell activation	↑ CRLS1, ↓ MCT1, ↑ SDHA/SDHB	(63)
	↑SIRT2, deacetylation of NF-κB p65; ↓nuclear translocation, restore the activity of CYP3A	↓ NF-κB signaling pathway, ↓ PARP-1,↑ NAD+	(14)
	↑Activation of FXR regulates bile acid metabolism; ↓ HSC activation, and proliferation	↑ Cyp7a1,↑ Sult2a8,↓ Slc51b/OSTβ	(64)
	↑ AMPK signaling pathway; ↓ TGF-β1/Smad2/3 pathway; ↓liver inflammation and fibrosis	↑ AMPK signaling pathway,↓ TGF-β1/Smad2/3 pathway	(66)
	Blocks the oxidative stress activation loop from hepatocytes to HSCs, and exerts synergistic anti-fibrotic effects when combined with GS-9973	↓ ROS	(67)
	Synergistic anti-fibrotic effect in combination with the SYK inhibitor GS-9973	↓SYK kinase	(68)
Gastritis	↓ Phosphorylation and nuclear translocation of NF-κB p65 and STAT3 block their synergistic pro-inflammatory effect	↓ COX-2,↓ iNOS	(69)
Ulcerative colitis	Remodel the gut microbiome, ↑ (R)-2,3-dihydroxyisovaleric acid; the metabolite targets GAT-3; ↑ GAT-3/RARβ/RORγt signaling axis; ↓ pro-inflammatory Th17 cell differentiation	↑ GAT-3/RARβ/RORγt signaling axis	(70)
Intestinal tumor	For normal intestinal epithelial cells:↑mitochondrial membrane, metabolism, and proliferation	↑ TGF-β,↓ TNF-α	(17)
	For intestinal tumor cells: ↓proliferation, induce apoptosis	↓ IL-1, ↓ IL-6, ↓ TGF-β	(17)
Gut-liver axis-related injury	↑ Solubility of the HP-β-CD complex restores intestinal flora and barrier function, ↓ intestinal-derived liver inflammation, and lipid accumulation		(71)

“↑” represents silybin’s promoting effect. “↓” represents silybin’s inhibiting effect.

## 7 The anti-inflammatory mechanism of silybin in metabolic syndrome

### 7.1 The impact on diabetes

Silybin exerts its core anti-inflammatory mechanism in diabetes and its complications through multi-target synergistic action. Studies have shown that silybin can activate the core insulin signaling pathway PI3K-Akt, significantly enhancing cell survival in the inflammatory microenvironment; at the same time, it can maintain the stable expression of estrogen receptor α (ERα) and β (ERβ), and activation of either receptor can induce protective autophagy, while also inhibiting the mTOR pathway to form a synergistic effect, thereby upregulating insulin signaling to protect islet β cells and inhibit their apoptosis (17). In addition, silybin can directly reduce the levels of key pro-inflammatory factors such as TNF-α, IL-1β, and IL-6, activate the AMPK pathway, and inhibit the transmission of downstream TGF-β1/Smad pro-fibrotic signals through this pathway, further strengthening its anti-inflammatory and anti-complication effects (66). In the context of hepatic metabolism, *in vitro* and *in vivo* experiments have found that silybin improves fatty liver and insulin resistance primarily by targeting and downregulating the expression of the microRNA miR-122. This coordinates the regulation of key enzymes in lipid metabolism: it inhibits the expression of lipogenic enzymes (FAS and ACC) while upregulating the rate-limiting enzyme for fatty acid β-oxidation, CPT1A, thus correcting lipid metabolic imbalance, reducing hepatic lipid deposition, and improving insulin resistance from the metabolic source (72). Notably, derivatives of silybin have been designed to enhance its efficacy by targeting specific deficiencies in diabetes. For instance, a novel silybin derivative, Bin-4, aimed at correcting hydrogen sulfide (H_2_S) deficiency in type 2 diabetes, promotes the nuclear translocation of the antioxidant transcription factor Nrf2 by upregulating the PI3K/AKT/GSK-3β signaling pathway. This protects GLUTag cells (which secrete GLP-1) from oxidative damage and apoptosis. Moreover, Bin-4 can directly activate estrogen receptor α, increasing the secretion level of GLP-1 by approximately 30%. *In vivo*, Bin-4 significantly reduces blood glucose and increases insulin and GLP-1 levels through this synergistic mechanism, providing a theoretical basis for the design of gas signaling molecule-based anti-diabetic drugs (73).

### 7.2 The impact on fat metabolism

Recent experiments have found that silybin directly downregulates the expression of key pro-inflammatory factors (such as TNF-α and IL-1β) by inhibiting the NF-κB pathway, thereby reducing macrophage recruitment and inflammation mediator production in adipose tissue, and helping to restore the expression of the anti-inflammatory adipokine adiponectin. At the same time, it activates the Nrf2 antioxidant defense pathway, synergistically enhancing the anti-inflammatory and anti-oxidation capabilities of cells. These systemic effects are crucial in the liver, where the aforementioned anti-inflammatory and anti-oxidation actions are further translated into direct improvements in lipid metabolism: by downregulating the expression of the lipid uptake receptor CD36 to inhibit lipid influx, and by reducing *de novo* lipogenesis through the inhibition of the expression of genes such as fatty acid synthase (FAS) and acetyl-CoA carboxylase (ACC), thereby improving fatty liver and systemic glucose-lipid metabolic homeostasis through multiple targets (74). Importantly, by ameliorating the metabolic and inflammatory microenvironment, silybin effectively blocks the liver carcinogenic process driven by obesity. Its core mechanism lies in inhibiting the phosphorylation of the Erk signaling pathway, thereby restraining the abnormal proliferation and invasion of hepatocytes. Silybin can simultaneously inhibit the aforementioned pathways and factors, reducing the protein levels of pErk, FASN, IL-6, and IL-1β, thereby decreasing the production of ROS and significantly inhibiting the secretion of matrix metalloprotein-9 (MMP-9) induced by obesity. Therefore, silybin provides an important molecular intervention strategy to delay the progression of non-alcoholic fatty liver disease to hepatic carcinoma by synergistically regulating multiple pathways of “Erk-proliferation,” “FASN-lipid metabolism,” and “IL-6/IL-1β-inflammation.” (75). Beyond the general mechanisms of silybin, research on specific derivatives has revealed unique actions. The core of the hepatoprotective effect of the derivative silybin A lies in driving the “lipid category conversion,” which transforms storage triglycerides into structural/functional phospholipids. Its molecular mechanism involves the bidirectional regulation of lipid metabolism-related enzymes: on one hand, it inhibits phospholipid degradation and promotes phospholipid synthesis, while on the other hand, it downregulates the biosynthesis of triglycerides. The structure-activity relationship indicates that its 1,4-benzodioxole ring configuration is crucial for reducing triglycerides, while the saturated 2,3-bond of the flavanol part is essential for phospholipid accumulation. Through this lipid remodeling and membrane system expansion, silybin A enhances the liver’s biotransformation capacity, thereby protecting mild metabolic disorders (76). Research has found that in a model of metabolic dysfunction-associated steatotic liver disease (MASLD), the level of the key microbial bile acid metabolite 7-keto-deoxycholic acid (7-KDCA)—a confirmed FXR antagonist—is elevated. Silybin and isosilybin A may reduce the production of 7-KDCA by directly targeting FXR and potentially modulating the gut microbiota, thereby providing negative feedback regulation of the FXR signaling pathway. Since FXR is a key nuclear receptor regulating glucose and lipid homeostasis, its activation helps improve metabolic inflammation. Therefore, this FXR-targeted action represents an important mechanism through which silybin and isosilybin A improve metabolic disorders, including insulin resistance (77). In the non-alcoholic steatohepatitis (NASH) model, silybin can inhibit the formation of neutrophil extracellular traps (NETs), which are key factors that trigger and exacerbate macrophage inflammatory response. Silybin significantly alleviated liver inflammation by inhibiting NETs, revealing its dual mechanism of regulating fat metabolism and inflammation (78).

**Figure 4:**
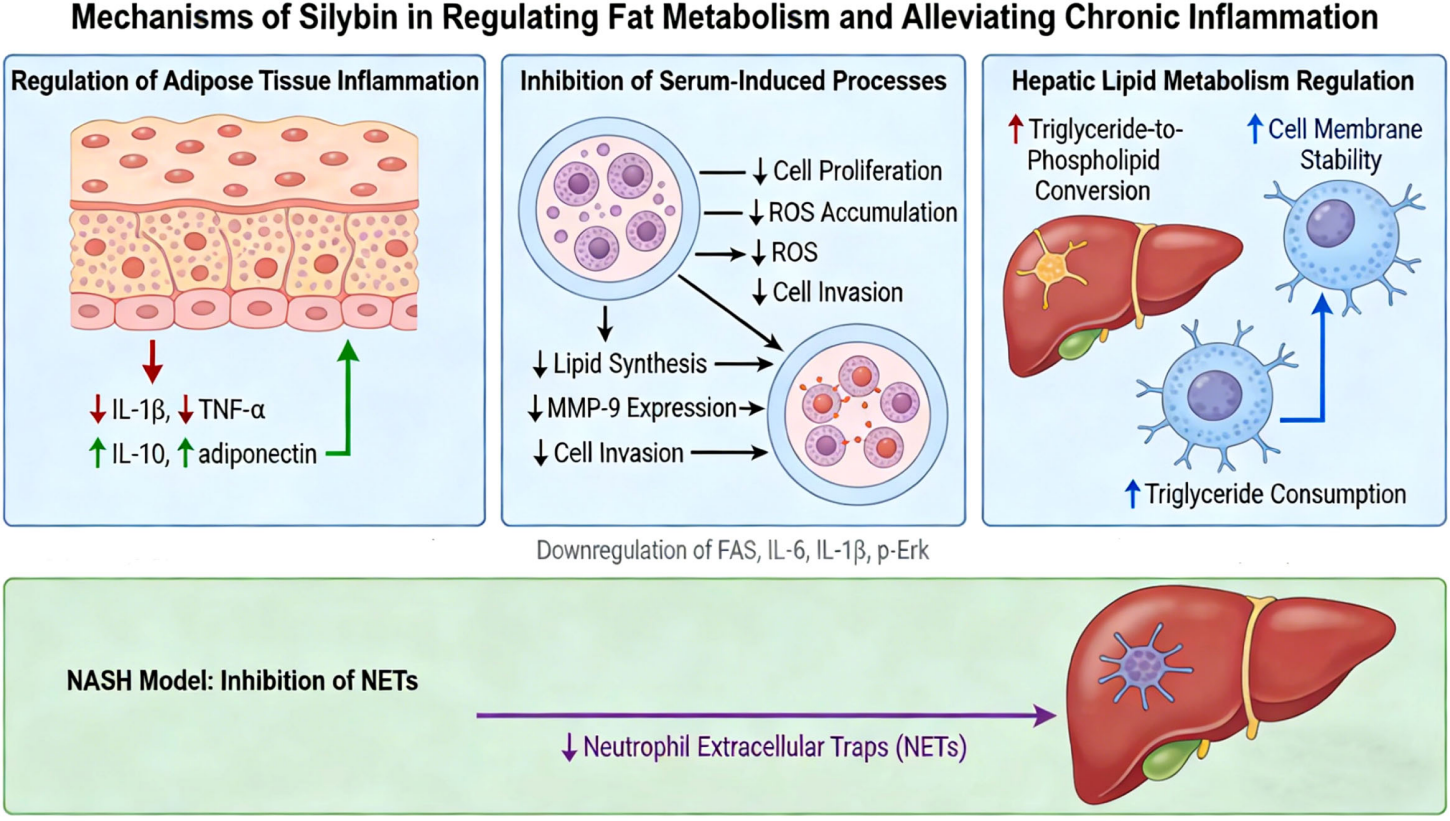
Mechanisms of silybin in regulating fat metabolism. The schematic illustrates the multifaceted regulatory effects of silybin on metabolic and inflammatory pathways. In adipose tissue, silybin modulates inflammation by downregulating pro-inflammatory cytokines (IL-1β, TNF-α) and upregulating anti-inflammatory mediators (IL-10, adiponectin). Silybin also inhibits serum-induced processes, including lipid synthesis, MMP-9 expression, and cell invasion. In the liver, silybin promotes hepatic lipid metabolism regulation by facilitating triglyceride-to-phospholipid conversion, enhancing cell membrane stability, and increasing triglyceride consumption. These effects are associated with the downregulation of key lipogenic and inflammatory markers, including FAS, IL-6, IL-1β, and p-Erk. Additionally, in a non-alcoholic steatohepatitis (NASH) model, silybin inhibits the formation of neutrophil extracellular traps (NETs), further contributing to the alleviation of chronic inflammation and metabolic dysfunction.

Silybin improves insulin sensitivity, protects pancreatic β-cell function, regulates adipose tissue inflammation, and liver lipid metabolism through the activation of antioxidant enzymes, inhibition of the NF-κB pathway, and multiple metabolic regulatory effects, collectively exerting a comprehensive benefit in improving insulin resistance and dysregulation of glucose and lipid metabolism (**Table 5**) (**Figure 4**).

**Table 5:** The role of silybin in metabolic syndrome.

Diseases	Key associated effect	Regulated factors	Reference
Diabetes mellitus and its complications	↑ Activate the PI3K-Akt signaling pathway, stabilize ERα/ERβ expression, induce protective autophagy; ↓ mTOR signaling pathway, and protect pancreatic islet β-cells	↑PI3K-Akt signaling pathway, ↓ mTOR signaling pathway	(17)
	↓ pro-inflammatory cytokine levels;↑ AMPK signaling pathway; ↓TGF-β1/Smad pro-fibrotic signaling pathway	↓ TNF-α,IL-1β,IL-6,↑ AMPK signaling pathway; ↓ TGF-β1/Smad pro-fibrotic signaling pathway	(66)
	↓miR-122 regulates key enzymes in lipid metabolism	↓ miR-122, ↓ FAS, ↓ ACC, ↑ CPT1A	(72)
	↑ PI3K/AKT/GSK-3β signaling pathway; ↑ Nrf2 nuclear translocation; ↑ Erα; ↑ GLP-1 secretion	↑ PI3K/AKT/GSK-3β signaling pathway, ↑ Nrf2 nuclear translocation, ↑ GLP-1	(73)
Abnormal lipid metabolism/fatty liver disease	↓ NF-κB signaling pathway; ↓ adipose tissue inflammation; ↑ Nrf2 antioxidant pathway, restore adiponectin; ↓ lipid uptake and synthesis; ↑ glucose and lipid metabolism	↓ NF-κB signaling pathway, ↑ Nrf2 antioxidant pathway,↓ CD36, ↓ FAS, ↓ ACC	(74)
	↓ phosphorylation of the Erk signaling pathway, block obesity-associated liver carcinogenesis	↓ phosphorylation of the Erk signaling pathway,↓ FASN, ↓ IL-6, ↓ IL-1β	(75)
	↑ lipid class switching, regulates triglyceride and phospholipid metabolism		(76)
MASLD	target FXR, regulate gut microbiota and 7-KDCA production, and negatively regulate the FXR pathway		(77)
NASH	↓ NETs formation, macrophage inflammation	↓ NETs	(78)

“↑” represents silybin’s promoting effect. “↓” represents silybin’s inhibiting effect.

## 8 The role of silybin in other diseases

### 8.1 Research progress on the application in dermatology

**Figure 5:**
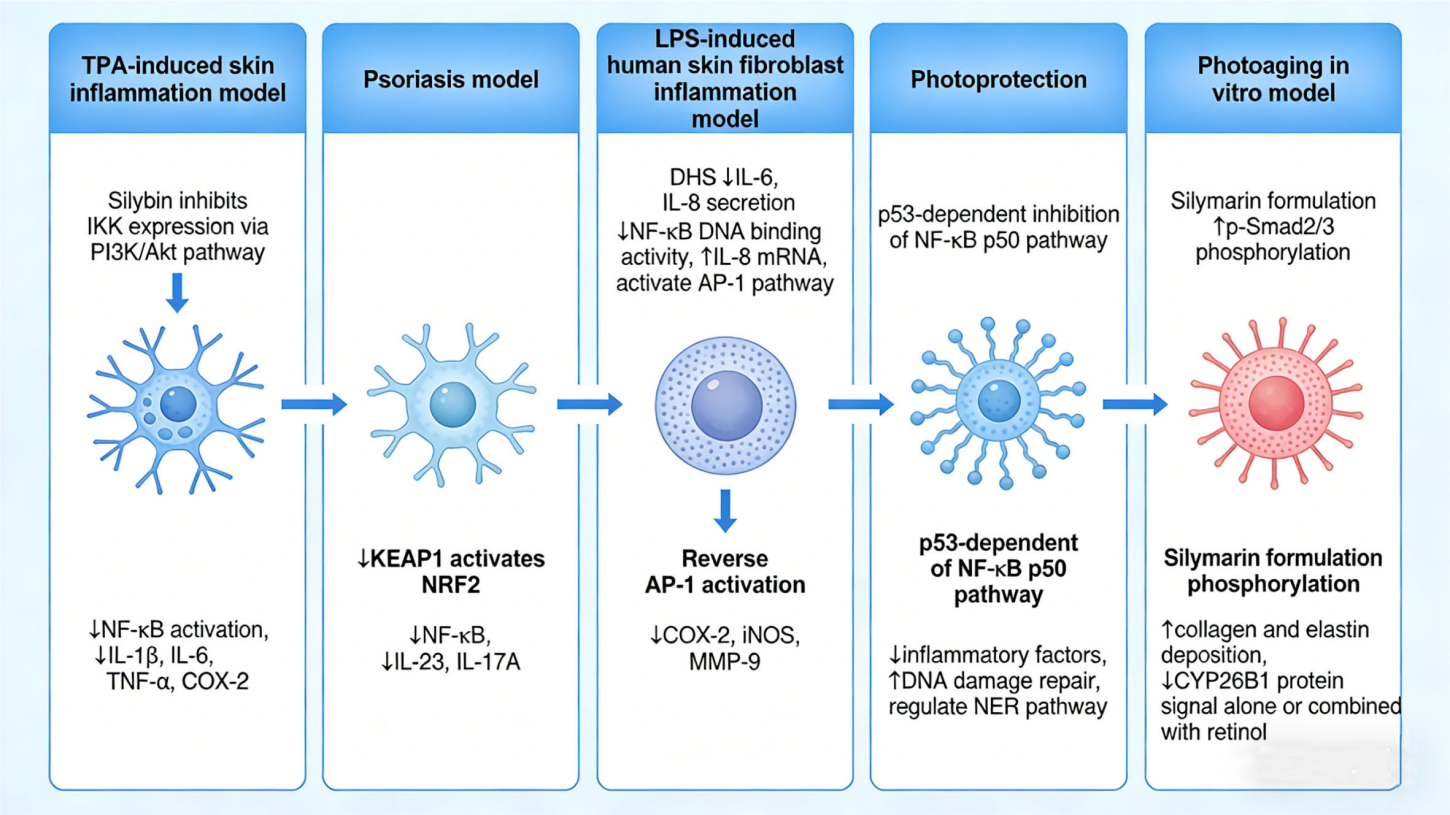
Multifaceted effects of silybin and silymarin on skin inflammation. The schematic summarizes the mechanisms identified in various skin models. In the TPA-induced skin inflammation model, silybin inhibits IKK expression via the PI3K/Akt pathway, leading to suppression of NF-κB activation and downregulation of pro-inflammatory cytokines (IL-1β, IL-6, TNF-α) and COX-2. In a psoriasis model, silybin reduces NF-κB activity and decreases IL-23 and IL-17A levels. In LPS-induced human skin fibroblast inflammation, DHS (dehydrosilybin) attenuates IL-6 and IL-8 secretion, inhibits NF-κB DNA binding activity, increases IL-8 mRNA, and activates the AP-1 pathway. Photoprotection is mediated through p53-dependent inhibition of the NF-κB p50 pathway. Additionally, silymarin formulation enhances p-Smad2/3 phosphorylation; JKEAP1 activates NRF2, resulting in decreased NF-κB, IL-23, and IL-17A. Reversal of AP-1 activation leads to downregulation of COX-2, iNOS, and MMP-9. Furthermore, silymarin formulation promotes collagen and elastin deposition and reduces CYP26B1 protein signal, both alone and in combination with retinol, indicating potential anti-aging and matrix-stabilizing effects.

The skin, as the largest organ of the human body, plays a key role in the occurrence and maintenance of inflammation due to its complex immune microenvironment and barrier function. Studies have shown that skin inflammation is not limited to the local area but may trigger systemic inflammatory responses, further leading to the occurrence of other systemic diseases. For example, the severity of skin lesions in psoriasis patients is associated with the levels of systemic inflammatory markers and increases the risk of cardiovascular disease, suggesting that skin inflammation can serve as an important mediating factor for systemic inflammation (79). Furthermore, taking Omenn syndrome as an example, intestinal inflammation can promote the development of skin inflammation by regulating skin chemokines and T cell recruitment, highlighting the important role of the gut-skin axis in inflammatory skin diseases (80). In the skin inflammation model induced by 12-O-tetradecanoylphorbol-13-acetate (TPA), silybin not only inhibits the expression of IL-1β, IL-6, TNF-α, and COX-2, but also suppresses the expression of IκB kinase (IKK) by inhibiting the PI3K/Akt signaling pathway, thereby inhibiting the activation of NF-κB (81). In psoriasis models, this effect is also associated with the downregulation of KEAP1 and activation of NRF2, which synergistically inhibit NF-κB and reduce the downstream factors IL-23 and IL-17A (82). In a sulfur mustard (HD)-induced skin injury model, silybin not only completely reverses the activation of activator protein 1 (AP-1) but also acts synergistically to inhibit NF-κB. This dual inhibition leads to the downregulation of key pro-inflammatory mediators, including COX-2, iNOS, and matrix metalloproteinase-9 (MMP-9) (83). Research has also found that in the inflammation model of human dermal fibroblasts induced by lipopolysaccharide (LPS), the derivative 2,3-dehydrosilybin (DHS) can inhibit the secretion of IL-6 and IL-8, suppress NF-κB DNA binding activity, and alleviate LPS-induced excessive inflammation. However, it upregulates IL-8 mRNA and activates the AP-1 pathway, which may lead to the potential synthesis reserve of proinflammatory effectors, and the sustained activation of AP-1 can induce apoptosis, disrupting the normal repair of skin tissue, suggesting that this drug is not suitable for long-term application in the treatment of skin inflammation (84). In the context of photoprotection, the anti-inflammatory effect of silybin applied locally is an important component of its mechanism against skin injury caused by ultraviolet B (UVB). The anti-inflammatory effect of silybin mainly inhibits the NF-κB p50 pathway in a p53-dependent manner, thereby downregulating COX-2, iNOS, TNF-α, IL-6, etc. It can also indirectly reduce inflammation by promoting DNA damage repair through the activation of p53, while the regulation of the nucleotide excision repair pathway also provides an auxiliary effect for anti-inflammation (85). In the *in vitro* model of photoaging, it was found that the addition of silymarin preparation further increased the phosphorylation level of p-Smad2/3 and enhanced the deposition of collagen (especially type XVII) and elastin. The use of silymarin alone or in combination with retinol can reduce the CYP26B1 protein signal, which may indirectly promote the anti-inflammatory effect and improve the extracellular matrix (ECM) by regulating the clearance of retinol through CYP26B1 (86). Collectively, these findings provide experimental evidence for the clinical application of silybin in chronic inflammatory skin diseases in the future(**Figure 5**).

### 8.2 Mechanism of action on ocular inflammation

Inflammation is also a core mechanism in the pathologic processes of various eye diseases. In the glaucoma-related HTM cell fibrosis model, silybin extensively regulates the expression profiles of coding genes (mRNA) and non-coding RNAs (LncRNA, CircRNA, miRNA), affecting multiple core signaling pathways such as TGF-β, PI3K-Akt, and NF-κB, and may ultimately achieve the effects of inhibiting extracellular matrix deposition, anti-fibrosis, and regulating inflammatory responses by constructing a ceRNA network (87). In the context of treating age-related macular degeneration (AMD), the compound silybin exerts an anti-inflammatory effect primarily by inhibiting the PI3K/Akt/mTOR/p70S6K signaling pathway (88). This inhibition leads to a decrease in the levels of hypoxia-inducible factor-1α (HIF-1α), thereby reducing the production of vascular endothelial growth factor (VEGF). Concurrently, silybin regulates the degradation of HIF-1α by increasing the levels of prolyl hydroxylase PHD2 and reducing the interaction between HIF-1α and the VHL protein (89). Together, these mechanisms work to inhibit pathological neovascularization and increased vascular permeability, resulting in anti-inflammatory and anti-edema effects (90).

### 8.3 Protective effect against dental inflammation

The occurrence and transmission of periodontal diseases are mediated by dysbiosis of the oral microbiota (dental plaque), which then interacts with the host’s immune defenses, leading to inflammation and disease (91).In both *in vivo* periodontitis models and *in vitro* LPS-stimulated human periodontal ligament cell (hPDLCs) models, it was found that silybin primarily exerts its core anti-inflammatory effect by downregulating the NF-κB and NLRP3 inflammasome signaling axis, thereby effectively inhibiting the expression of key pro-inflammatory cytokines TNF-α, IL-1β, and IL-6 in the periodontium. Simultaneously, it alleviates oxidative stress by activating the Nrf2 antioxidant pathway, indirectly breaking the vicious cycle of mutual exacerbation between inflammation and oxidative damage, synergistically enhancing the protective effect against periodontitis (92).

Silybin can exert anti-inflammatory effects in skin diseases, ocular diseases, and oral diseases by regulating signaling pathways such as NF-κB and AP-1, modulating immune cells, and other mechanisms, thereby alleviating tissue damage and improving disease-related symptoms (**Table 6**).

**Table 6:** The anti-inflammatory mechanism of silybin in other diseases.

Diseases	Key associated effect	Regulated factors	Reference
TPA-induced skin inflammation	↓ PI3K/Akt signaling pathway; ↓ NF-κB activation	↓ PI3K/Akt signaling pathway, ↓ NF-κB activation, ↓ IL-1β, ↓ IL-6, ↓ TNF-α, ↓ COX-2, ↓ IKK	(81)
Psoriasis	↓ PI3K/Akt/mTOR signaling pathway; ↓ activation of NF-κB	↓ PI3K/Akt/mTOR signaling pathway,↓ KEAP 1, ↑ NRF 2, ↓ NF-κB signaling pathway,↓ IL-23, ↓ IL-17 A	(82)
HD-induced skin injury	↓ AP-1/NF-κB dual signaling pathways; ↓ multiple pro-inflammatory mediators	↓ COX-2, ↓ iNOS, ↓ MMP-9	(83)
LPS-induced inflammation in human dermal fibroblasts	↓ NF-κB DNA-binding activity	↓ IL-6,↓ IL-8,	(84)
	↑ IL-8 mRNA; ↑ AP-1 signaling pathway	↑ AP-1 signaling pathway	
UVB-induced skin photodamage	↓ NF-κB p50 pathway; ↑ DNA repair	p53, ↓ COX-2, ↓ iNOS, ↓ TNF-α, ↓ IL-6	(85)
Photoaging	↑ Phosphorylation of p-Smad2/3; ↑ collagen, and elastin deposition	↓ CYP26B1, ↑ ECM	(86)
Glaucoma	Regulate the expression profiles of coding genes and non-coding RNAs, construct a ceRNA network, ↓ and extracellular matrix deposition, ↓ and fibrosis	↓ TGF-β, ↓ PI3K-Akt signaling pathway, ↑ NF-κB signaling pathway	(87)
AMD	↓ PI3K/Akt/mTOR/p70S6K signaling pathway; ↓ HIF-1α; ↓ VEGF	↓ PI3K/Akt/mTOR/p70S6K signaling pathway, ↓ HIF-1α, ↓ VEGF	(88)
	↑ PHD2; ↓ interaction between HIF-1α and VHL; ↑ degradation of HIF-1α	↑ PHD2	(88, 89)
Periodontitis	↓ NF-κB/NLRP3 inflammasome signaling axis, ↓ pro-inflammatory cytokines; ↑ Nrf2 antioxidant pathway; ↓ oxidative stress; vicious cycle between inflammation and oxidative damage	↓ NF-κB/NLRP3 inflammasome signaling axis,↓ TNF-α, ↓ IL-1β, ↓ IL-6, ↑ Nrf2 antioxidant pathway,	(92)

“↑” represents silybin’s promoting effect. “↓” represents silybin’s inhibiting effect.

## 9 Recent advances in silybin derivatives

### 9.1 Main structural modification sites and their association with anti-inflammatory activity

Esterification or etherification modification of the hydroxyl group at the C-7 position of silybin, such as linking amino acids or short-chain fatty acids, is an effective means to enhance its lipophilicity and cell membrane permeability, which helps to strengthen its anti-inflammatory activity within cells (28). Introducing hydrophilic groups, such as sulfonic acid or phosphate groups, at the C-23 position aims to improve its inherent defect of poor water solubility. This modification may alter its interaction pattern with cytoplasmic signaling proteins (such as the IKK complex), thereby more precisely regulating the inflammatory pathway (13). Additionally, structural modifications to the A and E rings, such as introducing halogens or methoxy groups, can change the electron cloud distribution and spatial conformation of the molecule. This change directly affects its binding affinity with key target proteins such as Keap1 or NF-κB, thereby regulating the expression of downstream inflammatory factors (93). These rational designs based on structure-activity relationships provide a solid chemical foundation for the development of highly active silybin derivatives.

### 9.2 Design of novel derivatives based on new mechanisms and technologies

Designing selective inhibitors for specific inflammatory targets is a current important direction. For example, derivatives aimed at inhibiting targets such as COX-2, 5-LOX, or the NLRP3 inflammasome are under development, to enhance anti-inflammatory efficacy while reducing potential side effects (8). The prodrug strategy, by designing derivatives that can be activated by enzymes specific to inflammatory sites (such as matrix metalloproteinases or esterases), enables targeted release and accumulation of the drug, thereby improving the therapeutic index (20). To overcome the challenge of low bioavailability of silybin, formulation research combining nano-delivery systems is particularly active. Encapsulating silybin or its derivatives using nano-systems such as liposomes and polymer micelles can significantly enhance their solubility, stability, and targeting ability (94). For example, functionalized selenium nanoparticles can effectively inhibit the PI3K/AKT/NF-κB signaling pathway, demonstrating excellent anti-inflammatory and antioxidant effects in cellular models (29). These approaches, based on new mechanisms and technologies, are driving the development of silybin derivatives toward more efficient and precise therapeutic strategies.

## 10 Conclusion

As a multi-target natural compound, silybin offers novel therapeutic perspectives for inflammation-related diseases by simultaneously regulating inflammatory signaling pathways, alleviating oxidative stress, and modulating immune cell functions. A large number of basic and clinical studies have shown that silybin not only exhibits significant efficacy in alleviating inflammatory responses in liver diseases but also demonstrates broad anti-inflammatory potential in complex pathological environments such as metabolic syndrome and neuroinflammation. This multi-target and multi-pathway therapeutic characteristic highlights the unique value of silybin as a natural medicine in modern medicine.

The research progress of silybin reflects the current trend in the field of anti-inflammatory therapy, shifting from single-target to multi-target regulation. It achieves a synergistic anti-inflammatory effect by regulating key inflammatory signaling pathways such as NF-κB and MAPK, alleviating oxidative stress, inhibiting the release of inflammatory mediators, and improving immune cell functions. This multidimensional mode of action enhances treatment specificity and efficacy. Moreover, it mitigates the risks of drug resistance and side effects commonly associated with single-target therapies. However, there are still certain controversies regarding the specific molecular mechanisms of silybin in existing studies, as some research results vary in different disease models, suggesting that its mechanisms may be influenced by factors such as disease type, dosage, and administration methods. Therefore, how to reasonably integrate various research findings and balance the differences under different experimental conditions is key to promoting the clinical application of silybin.

Moreover, despite showing good anti-inflammatory effects in various disease models, the clinical translation of silybin still faces many challenges. Current clinical trials mostly focus on safety and preliminary efficacy evaluations, lacking systematic dosage optimization and long-term efficacy observations. Future studies should conduct large-scale, multi-center randomized controlled trials to explore the pharmacokinetic characteristics, dose-response relationships, and potential side effects of silybin, thereby formulating scientifically sound medication guidelines. At the same time, enhancing the bioavailability of silybin through modern drug delivery technologies will greatly facilitate its clinical promotion.

In summary, silybin and its derivatives, with their broad-spectrum anti-inflammatory mechanisms and good safety profiles, show potential to become important drugs in the field of anti-inflammatory therapy. They have good application prospects in various inflammation-related diseases, such as liver diseases, metabolic syndrome, autoimmunity, and neuroinflammation, and are expected to provide new strategies and ideas for clinical treatment. Future research should focus on in-depth analysis of mechanisms and accelerated clinical translation, promoting silybin from the laboratory to the clinic, thus providing more effective and safe treatment options for patients with inflammatory diseases. As seasoned researchers in the medical field, we look forward to silybin playing a greater role in the development of anti-inflammatory drugs, advancing the prevention and treatment of inflammation-related diseases to a new level.

## References

[1] Selc M Babelova A . Looking beyond silybin: the importance of other silymarin flavonolignans. Front Pharmacol. (2025) 16:1637393. doi: 10.3389/fphar.2025.1637393, PMID: 40761399 PMC12320500

[2] Wadhwa K Pahwa R Kumar M Kumar S Sharma PC Singh G . Mechanistic insights into the pharmacological significance of silymarin. Molecules (Basel Switzerland). (2022) 27:5327. doi: 10.3390/molecules27165327, PMID: 36014565 PMC9414257

[3] Kim S Chen J Cheng T Gindulyte A He J He S . PubChem 2025 update. Nucleic Acids Res. (2025) 53:D1516–d25. doi: 10.1093/nar/gkae1059, PMID: 39558165 PMC11701573

[4] Muchiri RN Van Breemen RB . Chemical standardization of milk thistle (Silybum marianum L.) extract using UHPLC-MS/MS and the method of standard addition. J Am Soc Mass Spectrometry. (2024) 35:1726–32. doi: 10.1021/jasms.4c00125, PMID: 38953246 PMC11311221

[5] Ranjan S Gautam A . Pharmaceutical prospects of Silymarin for the treatment of neurological patients: an updated insight. Front Neurosci. (2023) 17:1159806. doi: 10.3389/fnins.2023.1159806, PMID: 37274201 PMC10232807

[6] Mohammad Ali FJ Zare F Sakhteman A Bahadori S Seradj H Emami L . Molecular docking studies, DFT, and ADMET calculations of some flavonoids and their characteristic structural features involved in inhibition of pro-inflammatory enzymes. Natural product Res. (2025) 39:5289–99. doi: 10.1080/14786419.2024.2368748, PMID: 39049514

[7] Braunstein I Motohashi H Dallenga T Schaible UE Benhar M . Redox signaling in innate immunity and inflammation: focus on macrophages and neutrophils. Free Radical Biol Med. (2025) 237:427–54. doi: 10.1016/j.freeradbiomed.2025.06.006, PMID: 40484207

[8] Yang HL Shi XW . Silybin alleviates experimental autoimmune encephalomyelitis by suppressing dendritic cell activation and th17 cell differentiation. Front neurology. (2021) 12:659678. doi: 10.3389/fneur.2021.659678, PMID: 34557140 PMC8452861

[9] Berbudi A Khairani S Tjahjadi AI . Interplay between insulin resistance and immune dysregulation in type 2 diabetes mellitus: implications for therapeutic interventions. ImmunoTargets Ther. (2025) 14:359–82. doi: 10.2147/itt.S499605, PMID: 40196377 PMC11974557

[10] Pagano MT Fecchi K Pierdominici M Ortona E Peruzzu D . Human monocyte-derived dendritic cells are the pharmacological target of the immunosuppressant flavonoid silibinin. Int J Mol Sci. (2022) 23:10417. doi: 10.3390/ijms231810417, PMID: 36142329 PMC9499000

[11] Cui Y Zhi SM Ding PF Zhu T Chen XX Liu XZ . Silybin attenuates microglia-mediated neuroinflammation via inhibition of STING in experimental subarachnoid hemorrhage. Int immunopharmacology. (2025) 151:114305. doi: 10.1016/j.intimp.2025.114305, PMID: 39986195

[12] Benslama O Lekmine S Moussa H Tahraoui H Ola MS Zhang J . Silymarin as a therapeutic agent for hepatocellular carcinoma: A multi-approach computational study. Metabolites. (2025) 15:53. doi: 10.3390/metabo15010053, PMID: 39852395 PMC11767256

[13] Wu J Wen L Liu X Li Q Sun Z Liang C . Silybin: A review of its targeted and novel agents for treating liver diseases based on pathogenesis. Phytotherapy research: PTR. (2024) 38:5713–40. doi: 10.1002/ptr.8347, PMID: 39310970

[14] Zhang R Xu D Zhang Y Wang R Yang N Lou Y . Silybin restored CYP3A expression through the sirtuin 2/nuclear factor κ-B pathway in mouse nonalcoholic fatty liver disease. Drug Metab disposition: Biol fate chemicals. (2021) 49:770–9. doi: 10.1124/dmd.121.000438, PMID: 34183378

[15] Wen X Peng Y Zheng B Yang S Han J Yu F . Silybin induces endothelium-dependent vasodilation via TRPV4 channels in mouse mesenteric arteries. Hypertension research: Off J Japanese Soc Hypertension. (2022) 45:1954–63. doi: 10.1038/s41440-022-01000-4, PMID: 36056206

[16] Marková I Malínská H Hüttl M Miklánková D Oliyarnyk O Poruba M . The combination of atorvastatin with silymarin enhances hypolipidemic, antioxidant and anti-inflammatory effects in a rat model of metabolic syndrome. Physiol Res. (2021) 70:33–43. doi: 10.33549/physiolres.934587, PMID: 33453720 PMC8820516

[17] Zare Mehrjerdi P Asadi S Ehsani E Askari VR Baradaran Rahimi V . Silibinin as a major component of milk thistle seed provides promising influences against diabetes and its complications: a systematic review. Naunyn-Schmiedeberg’s Arch Pharmacol. (2024) 397:7531–49. doi: 10.1007/s00210-024-03172-x, PMID: 38801454

[18] Faixová D Ratvaj M Maruščáková IC Hrčková G Karaffová V Faixová Z . Silybin showed higher cytotoxic, antiproliferative, and anti-inflammatory activities in the caCo cancer cell line while retaining viability and proliferation in normal intestinal IPEC-1 cells. Life (Basel Switzerland). (2023) 13:492. doi: 10.3390/life13020492, PMID: 36836848 PMC9964225

[19] Romanucci V Pagano R Kandhari K Zarrelli A Petrone M Agarwal C . 7-O-tyrosyl silybin derivatives as a novel set of anti-prostate cancer compounds. Antioxidants (Basel Switzerland). (2024) 13:418. doi: 10.3390/antiox13040418, PMID: 38671866 PMC11047488

[20] Li X Zhu H Wang Y Zhang X Yang Z Yan X . Silymarin and silybin: rejuvenating traditional remedies with modern delivery strategies. Pharmaceutics. (2025) 17:1628. doi: 10.3390/pharmaceutics17121628, PMID: 41471139 PMC12736914

[21] Romanucci V Naletova I Pagano R Petrone M Attanasio F Zarrelli A . Design and synthesis of new Silybin-carnosine conjugates with antioxidant and cytoprotective effects in LPS-treated RAW264. 7 macrophages Bioorganic Chem. (2026) 168:109351. doi: 10.1016/j.bioorg.2025.109351, PMID: 41406786

[22] Křen V . Chirality matters: biological activity of optically pure silybin and its congeners. Int J Mol Sci. (2021) 22:7885. doi: 10.3390/ijms22157885, PMID: 34360650 PMC8346157

[23] Albassam AA Frye RF Markowitz JS . The effect of milk thistle (Silybum marianum) and its main flavonolignans on CYP2C8 enzyme activity in human liver microsomes. Chemico-biological interactions. (2017) 271:24–9. doi: 10.1016/j.cbi.2017.04.025, PMID: 28457856

[24] Wu S Chen G Chen EY Farshidpour LS Zhang Q Wang G . Core structure-activity relationship studies of 5,7,20-O-trimethylsilybins in prostate cancer cell models. Pharm (Basel Switzerland). (2023) 16:531. doi: 10.3390/ph16040531, PMID: 37111288 PMC10145751

[25] Chen J Li DL Xie LN Ma YR Wu PP Li C . Synergistic anti-inflammatory effects of silibinin and thymol combination on LPS-induced RAW264.7 cells by inhibition of NF-κB and MAPK activation. Phytomedicine: Int J phytotherapy phytopharmacology. (2020) 78:153309. doi: 10.1016/j.phymed.2020.153309, PMID: 32890914

[26] Jiang M He K Qiu T Sun J Liu Q Zhang X . Tumor-targeted delivery of silibinin and IPI-549 synergistically inhibit breast cancer by remodeling the microenvironment. Int J pharmaceutics. (2020) 581:119239. doi: 10.1016/j.ijpharm.2020.119239, PMID: 32194211

[27] Butanda-Nuñez A Rodríguez-Cortés O Ramos-Martínez E Cerbón MA Escobedo G Chavarría A . Silybin restores glucose uptake after tumour necrosis factor-alpha and lipopolysaccharide stimulation in 3T3-L1 adipocytes. Adipocyte. (2024) 13:2374062. doi: 10.1080/21623945.2024.2374062, PMID: 38953241 PMC11221471

[28] Dobiasová S Řehořová K Kučerová D Biedermann D Káňová K Petrásková L . Multidrug resistance modulation activity of silybin derivatives and their anti-inflammatory potential. Antioxidants (Basel Switzerland). (2020) 9:455. doi: 10.3390/antiox9050455, PMID: 32466263 PMC7278776

[29] Abd-Rabou AA Zoheir KMA Abdel-Aleem GA Osman A Elsabagh DT Kishta MS . Silymarin/silybin-functionalized selenium nanoparticles suppress lipopolysaccharide-induced inflammation via PI3K/AKT/NF-κB signaling pathway inhibition. Chem biodiversity. (2025) 22:e00990. doi: 10.1002/cbdv.202500990, PMID: 40566869

[30] Yıldız B Ergüç E Carpio LE Gozalbes R Ortiz-González C Orhan H . Silybin, silychristin and silydianin are potential novel plant-based Pannexin1 channel inhibitors. Int immunopharmacology. (2025) 164:115316. doi: 10.1016/j.intimp.2025.115316, PMID: 40773896

[31] Ashique S Mohanto S Kumar N Nag S Mishra A Biswas A . Unlocking the possibilities of therapeutic potential of silymarin and silibinin against neurodegenerative Diseases-A mechanistic overview. Eur J Pharmacol. (2024) 981:176906. doi: 10.1016/j.ejphar.2024.176906, PMID: 39154829

[32] Ma X Xia K Xie J Yan B Han X Li S . Treatment of idiopathic pulmonary fibrosis by inhaled silybin dry powder prepared via the nanosuspension spray drying technology. ACS Pharmacol Trans science. (2023) 6:878–91. doi: 10.1021/acsptsci.3c00033, PMID: 37325446 PMC10262316

[33] Spartali C Psarra AG Marras SI Tsioptsias C Georgantopoulos A Kalousi FD . Silybin-functionalized PCL electrospun fibrous membranes for potential pharmaceutical and biomedical applications. Polymers. (2024) 16:2346. doi: 10.3390/polym16162346, PMID: 39204566 PMC11359364

[34] Diukendjieva A Zaharieva MM Mori M Alov P Tsakovska I Pencheva T . Dual SMO/BRAF inhibition by flavonolignans from silybum marianum (†). Antioxidants (Basel Switzerland). (2020) 9:384. doi: 10.3390/antiox9050384, PMID: 32380762 PMC7278695

[35] Manivannan E Amawi H Hussein N Karthikeyan C Fetcenko A Narayana Moorthy NSH . Design and discovery of silybin analogues as antiproliferative compounds using a ring disjunctive - Based, natural product lead optimization approach. Eur J medicinal Chem. (2017) 133:365–78. doi: 10.1016/j.ejmech.2017.03.033, PMID: 28411546

[36] García-Viñuales S Ahmed R Sciacca MFM Lanza V Giuffrida ML Zimbone S . Trehalose conjugates of silybin as prodrugs for targeting toxic Aβ Aggregates. ACS Chem Neurosci. (2020) 11:2566–76. doi: 10.1021/acschemneuro.0c00232, PMID: 32687307

[37] Liu X Chen W Wang C Liu W Hayashi T Mizuno K . Silibinin ameliorates depression/anxiety-like behaviors of Parkinson’s disease mouse model and is associated with attenuated STING-IRF3-IFN-β pathway activation and neuroinflammation. Physiol behavior. (2021) 241:113593. doi: 10.1016/j.physbeh.2021.113593, PMID: 34536434

[38] Ramírez-Carreto RJ Zaldívar-Machorro VJ Pérez-Ramírez DJ Rodríguez-López BE Meza C García E . Oral administration of silybin protects against MPTP-induced neurotoxicity by reducing pro-inflammatory cytokines and preserving BDNF levels in mice. Mol neurobiology. (2023) 60:6774–88. doi: 10.1007/s12035-023-03485-7, PMID: 37480498 PMC10657796

[39] Liu X Liu W Wang C Chen Y Liu P Hayashi T . Silibinin attenuates motor dysfunction in a mouse model of Parkinson’s disease by suppression of oxidative stress and neuroinflammation along with promotion of mitophagy. Physiol behavior. (2021) 239:113510. doi: 10.1016/j.physbeh.2021.113510, PMID: 34181930

[40] Moosakhani B Taleb M Mahmoudi Eshkaftaki Z Nikfarjam N Serajian A Shahsavani MB . Inhibition of cytotoxic fibril formation of α-synuclein and human insulin by Silymarin from the Silybum marianum. PloS One. (2025) 20:e0320283. doi: 10.1371/journal.pone.0320283, PMID: 40315258 PMC12047757

[41] Khan S Khatri DK . In-silico screening to identify phytochemical inhibitor for hP2X7: A crucial inflammatory cell death mediator in Parkinson’s disease. Comput Biol Chem. (2025) 115:108285. doi: 10.1016/j.compbiolchem.2024.108285, PMID: 39615401

[42] Cebani L Mvubu NE . Can We Exploit Inflammasomes for Host-Directed Therapy in the Fight against Mycobacterium tuberculosis Infection? Int J Mol Sci. (2024) 25:8196. doi: 10.3390/ijms25158196, PMID: 39125766 PMC11311975

[43] Tiwari D Martineau AR . Inflammation-mediated tissue damage in pulmonary tuberculosis and host-directed therapeutic strategies. Semin Immunol. (2023) 65:101672. doi: 10.1016/j.smim.2022.101672, PMID: 36469987

[44] Rodríguez-Flores EM Mata-Espinosa D Barrios-Payan J Marquina-Castillo B Castañón-Arreola M Hernández-Pando R . A significant therapeutic effect of silymarin administered alone, or in combination with chemotherapy, in experimental pulmonary tuberculosis caused by drug-sensitive or drug-resistant strains: *In vitro* and *in vivo* studies. PloS One. (2019) 14:e0217457. doi: 10.1371/journal.pone.0217457, PMID: 31145751 PMC6542514

[45] Chang CJ Lin CF Chen BC Lin PY Chen CL . SHP2: The protein tyrosine phosphatase involved in chronic pulmonary inflammation and fibrosis. IUBMB Life. (2022) 74:131–42. doi: 10.1002/iub.2559, PMID: 34590785

[46] Ma P Li S Yang H Yuan J Zhang Z Li X . Comparative RNA-seq transcriptome analysis on pulmonary inflammation in a mouse model of asthma-COPD overlap syndrome. Front Cell Dev Biol. (2021) 9:628957. doi: 10.3389/fcell.2021.628957, PMID: 33869177 PMC8044804

[47] Zhao Y Zhou Y Gong T Liu Z Yang W Xiong Y . The clinical anti-inflammatory effects and underlying mechanisms of silymarin. iScience. (2024) 27:111109. doi: 10.1016/j.isci.2024.111109, PMID: 39507256 PMC11539592

[48] Lim JO Shin NR Seo YS Nam HH Ko JW Jung TY . Silibinin attenuates silica dioxide nanoparticles-induced inflammation by suppressing TXNIP/MAPKs/AP-1 signaling. Cells. (2020) 9:678. doi: 10.3390/cells9030678, PMID: 32164364 PMC7140632

[49] Speciale A Muscarà C Molonia MS Cimino F Saija A Giofrè SV . Silibinin as potential tool against SARS-Cov-2: In silico spike receptor-binding domain and main protease molecular docking analysis, and *in vitro* endothelial protective effects. Phytotherapy research: PTR. (2021) 35:4616–25. doi: 10.1002/ptr.7107, PMID: 33822421 PMC8251480

[50] Saadh MJ Muhammad FA Albadr RJ Sanghvi G Jyothi SR Kundlas M . Inflammasomes and cardiovascular disease: linking inflammation to cardiovascular pathophysiology. Scandinavian J Immunol. (2025) 101:e70020. doi: 10.1111/sji.70020, PMID: 40170223

[51] Sethwala AM Goh I Amerena JV . Combating inflammation in cardiovascular disease. Heart Lung circulation. (2021) 30:197–206. doi: 10.1016/j.hlc.2020.09.003, PMID: 33039279

[52] Goswami SK Ranjan P Dutta RK Verma SK . Management of inflammation in cardiovascular diseases. Pharmacol Res. (2021) 173:105912. doi: 10.1016/j.phrs.2021.105912, PMID: 34562603 PMC8541927

[53] Akhmerov A Parimon T . Extracellular vesicles, inflammation, and cardiovascular disease. Cells. (2022) 11:2229. doi: 10.3390/cells11142229, PMID: 35883672 PMC9320258

[54] Henein MY Vancheri S Longo G Vancheri F . The role of inflammation in cardiovascular disease. Int J Mol Sci. (2022) 23:12906. doi: 10.3390/ijms232112906, PMID: 36361701 PMC9658900

[55] Kadoglou NPE Panayiotou C Vardas M Balaskas N Kostomitsopoulos NG Tsaroucha AK . A comprehensive review of the cardiovascular protective properties of silibinin/silymarin: A new kid on the block. Pharm (Basel Switzerland). (2022) 15:538. doi: 10.3390/ph15050538, PMID: 35631363 PMC9145573

[56] Shen HR Wang ZY Shen Z Liu TT Guo YD Gao TL . Bacterial butyrate mediates the anti-atherosclerotic effect of silybin. BioMed Pharmacother. (2023) 169:115916. doi: 10.1016/j.biopha.2023.115916, PMID: 38000354

[57] Zhang J Shi Q Hu Y Li X . Silibinin augments the effect of clopidogrel on atherosclerosis in diabetic ApoE deficiency mice. Clin hemorheology microcirculation. (2022) 80:353–61. doi: 10.3233/ch-211279, PMID: 34602463

[58] Oates JC Russell DL Van Beusecum JP . Endothelial cells: potential novel regulators of renal inflammation. Am J Physiol Renal Physiol. (2022) 322:F309–F321. doi: 10.1152/ajprenal.00371.2021, PMID: 35129369 PMC8897017

[59] Saleem M Masenga SK Ishimwe JA Demirci M Ahmad T Jamison S . Recent advances in understanding peripheral and gut immune cell-mediated salt-sensitive hypertension and nephropathy. Hypertension (Dallas Tex: 1979). (2024) 81:436–46. doi: 10.1161/hypertensionaha.123.22031, PMID: 38164753 PMC10922672

[60] Wang M Chen J Zhang Z Wang T Zhao J Wang X . Silybin mitigates post-myocardial infarction heart failure in mice via modulation of HIF-1α-driven glycolysis and energy metabolism. Nutrients. (2025) 17:2800. doi: 10.3390/nu17172800, PMID: 40944191 PMC12430516

[61] Vargas-Mendoza N Morales-González Á Morales-Martínez M Soriano-Ursúa MA Delgado-Olivares L Sandoval-Gallegos EM . Flavolignans from silymarin as nrf2 bioactivators and their therapeutic applications. Biomedicines. (2020) 8:122. doi: 10.3390/biomedicines8050122, PMID: 32423098 PMC7277158

[62] Sui Z Li M Wang X Luo C Chen L Deng X . Discovery of a novel silybin derivative for the treatment of metabolic dysfunction-associated steatohepatitis. J medicinal Chem. (2025) 68:12786–99. doi: 10.1021/acs.jmedchem.5c00672, PMID: 40493802

[63] Yang X Lou Y Li H Ma Y Wang Z Aa J . Silybin inhibits succinate production and secretion in hepatocytes to reverse liver fibrosis. Arch pharmacal Res. (2025) 48:782–97. doi: 10.1007/s12272-025-01560-2, PMID: 40739373

[64] Liu X Xia N Yu Q Jin M Wang Z Fan X . Silybin meglumine mitigates CCl(4)-induced liver fibrosis and bile acid metabolism alterations. Metabolites. (2024) 14:556. doi: 10.3390/metabo14100556, PMID: 39452937 PMC11509150

[65] Li BY Xi Y Liu YP Wang D Wang C Chen CG . Effects of Silybum marianum, Pueraria lobate, combined with Salvia miltiorrhiza tablets on non-alcoholic fatty liver disease in adults: A triple-blind, randomized, placebo-controlled clinical trial. Clin Nutr ESPEN. (2024) 63:2–12. doi: 10.1016/j.clnesp.2024.06.003, PMID: 38879879

[66] Cai J Zhu Y Li X Deng G Han Y Yuan F . Liposomal silybin improves glucose and lipid metabolisms in type 2 diabetes mellitus complicated with non-alcoholic fatty liver disease via AMPK/TGF-β1/smad signaling. Tohoku J Exp Med. (2023) 261:257–65. doi: 10.1620/tjem.2023.J050, PMID: 37344419

[67] Zhang LF Deng WQ Huang QW Zhang JJ Wang Y Zhou TJ . Vicious cycle-breaking lipid nanoparticles remodeling multicellular crosstalk to reverse liver fibrosis. Advanced materials (Deerfield Beach Fla). (2024) 36:e2311474. doi: 10.1002/adma.202311474, PMID: 38194906

[68] Hu Q Liu M You Y Zhou G Chen Y Yuan H . Dual inhibition of reactive oxygen species and spleen tyrosine kinase as a therapeutic strategy in liver fibrosis. Free Radical Biol Med. (2021) 175:193–205. doi: 10.1016/j.freeradbiomed.2021.08.241, PMID: 34492311

[69] Cho K Lee HG Piao JY Kim SJ Na HK Surh YJ . Protective effects of silibinin on helicobacter pylori-induced gastritis: NF-κB and STAT3 as potential targets. J Cancer Prev. (2021) 26:118–27. doi: 10.15430/jcp.2021.26.2.118, PMID: 34258250 PMC8249208

[70] Yan B Zheng X Lu D Li T Chen X Shao Z . Silibinin-derived microbiota enrich (R)-2,3-dihydroxy-isovalerate and ameliorate colitis via the GAT-3/RARβ/RORγt axis. ISME J. (2025) 19:wraf175. doi: 10.1093/ismejo/wraf175, PMID: 40801260 PMC12448484

[71] Ren L Ma XL Wang HL Li R Cui JJ Yan PJ . Prebiotic-like cyclodextrin assisted silybin on NAFLD through restoring liver and gut homeostasis. J Controlled release: Off J Controlled Release Society. (2022) 348:825–40. doi: 10.1016/j.jconrel.2022.06.031, PMID: 35752255

[72] Yang L Liu Q Zhang H Wang Y Li Y Chen S . Silibinin improves nonalcoholic fatty liver by regulating the expression of miR−122: An *in vitro* and *in vivo* study. Mol Med Rep. (2021) 23:335. doi: 10.3892/mmr.2021.11974, PMID: 33760189 PMC7974327

[73] Li H Ma Q Li X Zheng C Lin L Gu J . Development of hydrogen sulfide-donating silybin derivatives with a type-2 diabetes mellitus-alleviating effect through improving intestinal L-cell functions. J medicinal Chem. (2025) 68:15520–42. doi: 10.1021/acs.jmedchem.5c00194, PMID: 40759002

[74] Alsaggar M Bdour S Ababneh Q El-Elimat T Qinna N Alzoubi KH . Silibinin attenuates adipose tissue inflammation and reverses obesity and its complications in diet-induced obesity model in mice. BMC Pharmacol toxicology. (2020) 21:8. doi: 10.1186/s40360-020-0385-8, PMID: 31973745 PMC6979281

[75] Miethe C Nix H Martin R Hernandez AR Price RS . Silibinin reduces the impact of obesity on invasive liver cancer. Nutr cancer. (2017) 69:1272–80. doi: 10.1080/01635581.2017.1367935, PMID: 29068700

[76] Koeberle SC Thürmer M Su F Werner M Grander J Hofer L . Silybin A from Silybum marianum reprograms lipid metabolism to induce a cell fate-dependent class switch from triglycerides to phospholipids. Theranostics. (2025) 15:2006–34. doi: 10.7150/thno.99562, PMID: 39897559 PMC11780512

[77] Yi M Manzoor M Yang M Zhang H Wang L Zhao L . Silymarin targets the FXR protein through microbial metabolite 7-keto-deoxycholic acid to treat MASLD in obese mice. Phytomedicine. (2024) 133:155947. doi: 10.1016/j.phymed.2024.155947, PMID: 39178642

[78] Wu J Zhang C He T Zhang S Wang Y Xie Z . Polyunsaturated fatty acids drive neutrophil extracellular trap formation in nonalcoholic steatohepatitis. Eur J Pharmacol. (2023) 945:175618. doi: 10.1016/j.ejphar.2023.175618, PMID: 36841284

[79] Svedbom A Mallbris L González-Cantero Á Playford M Wu C Mehta NN . Skin inflammation, systemic inflammation, and cardiovascular disease in psoriasis. JAMA Dermatol. (2025) 161:81–6. doi: 10.1001/jamadermatol.2024.4433, PMID: 39565616 PMC11579891

[80] Rigoni R Fontana E Dobbs K Marrella V Taverniti V Maina V . Cutaneous barrier leakage and gut inflammation drive skin disease in Omenn syndrome. J Allergy Clin Immunol. (2020) 146:1165–79.e11. doi: 10.1016/j.jaci.2020.04.005, PMID: 32311393 PMC7649331

[81] Liu W Li Y Zheng X Zhang K Du Z . Potent inhibitory effect of silibinin from milk thistle on skin inflammation stimuli by 12-O-tetradecanoylphorbol-13-acetate. Food Funct. (2015) 6:3712–9. doi: 10.1039/c5fo00899a, PMID: 26345246

[82] Kamel NM El-Sayed SS El-Said Y El-Kersh DM Hashem MM Mohamed SS . Unlocking milk thistle’s anti-psoriatic potential in mice: Targeting PI3K/AKT/mTOR and KEAP1/NRF2/NF-κB pathways to modulate inflammation and oxidative stress. Int immunopharmacology. (2024) 139:112781. doi: 10.1016/j.intimp.2024.112781, PMID: 39059101

[83] Tewari-Singh N Jain AK Inturi S Agarwal C White CW Agarwal R . Silibinin attenuates sulfur mustard analog-induced skin injury by targeting multiple pathways connecting oxidative stress and inflammation. PloS One. (2012) 7:e46149. doi: 10.1371/journal.pone.0046149, PMID: 23029417 PMC3459894

[84] Juráňová J Aury-Landas J Boumediene K Baugé C Biedermann D Ulrichová J . Modulation of skin inflammatory response by active components of silymarin. Molecules (Basel Switzerland). (2018) 24:123. doi: 10.3390/molecules24010123, PMID: 30598040 PMC6337225

[85] Rigby CM Roy S Deep G Guillermo-Lagae R Jain AK Dhar D . Role of p53 in silibinin-mediated inhibition of ultraviolet B radiation-induced DNA damage, inflammation and skin carcinogenesis. Carcinogenesis. (2017) 38:40–50. doi: 10.1093/carcin/bgw106, PMID: 27729375 PMC5219048

[86] Shen Y Shi M Ye Y Xu C Feng X Bi T . An innovative serum with retinol, hydroxypinacolone retinoate, peptides, and silybin improves mild photoaged facial skin in middle-aged chinese women. J cosmetic Dermatol. (2026) 25:e70627. doi: 10.1111/jocd.70627, PMID: 41450017 PMC12741608

[87] Liu J Wu X Guo J Zhou L Liu X Zhu L . Integrative analysis of mRNA, lncRNA, circRNA, and miRNA to investigate the anti-fibrotic activity of silibinin in TGF-β2-treated human trabecular meshwork cells. BMC Genomics. (2025) 26:814. doi: 10.1186/s12864-025-12027-5, PMID: 40999359 PMC12465171

[88] Lin CH Li CH Liao PL Tse LS Huang WK Cheng HW . Silibinin inhibits VEGF secretion and age-related macular degeneration in a hypoxia-dependent manner through the PI-3 kinase/Akt/mTOR pathway. Br J Pharmacol. (2013) 168:920–31. doi: 10.1111/j.1476-5381.2012.02227.x, PMID: 23004355 PMC3631380

[89] Sesso HD Rautiainen S Park SJ Kim E Lee IM Glynn RJ . Intake of blueberries, anthocyanins, and risk of eye disease in women. J Nutr. (2024) 154:1404–13. doi: 10.1016/j.tjnut.2024.02.028, PMID: 38432561 PMC11007733

[90] Klusek K Kijowska M Kiełbus M Sławińska J Kuźmiuk D Chorągiewicz T . The supportive role of plant-based substances in AMD treatment and their potential. Int J Mol Sci. (2025) 26:7906. doi: 10.3390/ijms26167906, PMID: 40869227 PMC12386429

[91] Kinane DF Stathopoulou PG Papapanou PN . Periodontal diseases. Nat Rev Dis primers. (2017) 3:17038. doi: 10.1038/nrdp.2017.38, PMID: 28805207

[92] Li X Zhou R Han Y Zeng J Shi L Mao Y . Silibinin attenuates experimental periodontitis by downregulation of inflammation and oxidative stress. Oxid Med Cell longevity. (2023) 2023:5617800. doi: 10.1155/2023/5617800, PMID: 36846719 PMC9946757

[93] Wei RR Ma QG . Flavonolignan 2, 3-dehydroderivatives from Oenanthe javanica and their anti inflammatory activities. Z fur Naturforschung C J biosciences. (2021) 76:459–65. doi: 10.1515/znc-2021-0001, PMID: 34002579

[94] Dsouza MV Dodamani S Kurangi B Shetti P Gudasi S . Advanced HPTLC method development for silibinin analysis in nanoformulated scaffolds: A box-behnken approach. Phytochemical analysis: PCA. (2025) 36:866–75. doi: 10.1002/pca.3474, PMID: 39520204

